# High-resolution near real-time drought monitoring in South Asia

**DOI:** 10.1038/sdata.2017.145

**Published:** 2017-10-03

**Authors:** Saran Aadhar, Vimal Mishra

**Affiliations:** 1 Civil Engineering, Indian Institute of Technology (IIT), Gandhinagar, India

**Keywords:** Hydrology, Natural hazards

## Abstract

Drought in South Asia affect food and water security and pose challenges for millions of people. For policy-making, planning, and management of water resources at sub-basin or administrative levels, high-resolution datasets of precipitation and air temperature are required in near-real time. We develop a high-resolution (0.05°) bias-corrected precipitation and temperature data that can be used to monitor near real-time drought conditions over South Asia. Moreover, the dataset can be used to monitor climatic extremes (heat and cold waves, dry and wet anomalies) in South Asia. A distribution mapping method was applied to correct bias in precipitation and air temperature, which performed well compared to the other bias correction method based on linear scaling. Bias-corrected precipitation and temperature data were used to estimate Standardized precipitation index (SPI) and Standardized Precipitation Evapotranspiration Index (SPEI) to assess the historical and current drought conditions in South Asia. We evaluated drought severity and extent against the satellite-based Normalized Difference Vegetation Index (NDVI) anomalies and satellite-driven Drought Severity Index (DSI) at 0.05°. The bias-corrected high-resolution data can effectively capture observed drought conditions as shown by the satellite-based drought estimates. High resolution near real-time dataset can provide valuable information for decision-making at district and sub-basin levels.

## Background & Summary

Drought is one of the most complex natural disasters, which is often difficult to identify, (including the start, end, intensity, and extent), predict, and mitigate. In South Asia, increasing population and frequent droughts have been significant factors in furthering the water crisis and food scarcity. The drought of 2002 was among the most severe events, affecting around 300 million people in India^1^. The South Asian region faced multiple severe and long-lasting droughts, which posed tremendous impacts on growing economies of the region. India, Pakistan, and Sri Lanka have reported frequent droughts (once in every three years) in the last decades. In India, about 330 million people were affected due to the 2014–15 drought which had a return period of 542 years^2^ and led to a severe water shortage. Pakistan also experienced many droughts, and the 1999–2000 (continuing up to 2002) drought affected about 3.3 million people and resulted in a negative growth of 2.6% in the agriculture sector^3^. Due to severe droughts, the Pakistan government announced emergency conditions^4^. In Bangladesh, 19 droughts occurred between 1960 and 1991. On an average, a severe drought occurs in Bangladesh once in every 2.5 years^5^ and affects 53% of the population and 40% of crop production. Currently (in 2017), South India and Sri Lanka are facing a severe drought that has already affected more than 0.2 million people.

A high-resolution near real-time drought monitoring system is required for South Asia to assist policy makers and water managers and to minimize the detrimental impacts of water and food scarcity. Drought information at district to village level is needed for decision making and adaptation^6,7^ (GWP Assessment Report, 2014). In South Asia, only India has a near real-time drought monitoring system, however, at a coarser spatial resolution (0.25°). The Indian experimental drought monitor system^8^ provides near real-time (1-day lag) drought information. The India Meteorological Department (IMD) (www.imdpune.gov.in) also provides drought information in India, however, at monthly scale and much coarser spatial resolution. The Pakistan Meteorological Department (PMD) (http://www.pmd.gov.pk/) provides monthly drought information using rain gauge station data and satellite-based vegetation index. The satellite-based near real-time drought monitoring and early warning system (DMEWS, http://wtlab.iis.u-tokyo.ac.jp/DMEWS/) for Asian Pacific countries^9^ provide drought warning at the state level. Drought monitoring systems at global scales^10^ have also been valuable for decision making at national levels.

While existing drought monitoring systems^8–10^ provide information at the regional and global scales, those are unable to help in decision making at the local scale (sub-basin or district levels) due to their coarse spatial resolution. A high-resolution near real-time precipitation and air temperature (maximum and minimum) dataset is required for drought monitoring at the district and sub-basin levels in South Asia. Here we use Climate Hazards Group of Infra-Red Precipitation with Stations (CHIRPS^11^) and maximum and minimum air temperatures from Global Ensemble Forecast System (GEFS) reforecast version 2 (ref. 12) from 1981 onwards to develop a bias-corrected precipitation and temperature product that can be used for drought monitoring in the South Asia. We used the bias corrected datasets to estimate Standardized Precipitation Index^13^ (SPI) and Standardized Precipitation Evapotranspiration Index^14^ (SPEI) at 0.05° spatial resolution. The drought information at high resolution can be useful for policy makers, stakeholders, and water managers to in decision-making and as well as in climate change adaptation.

## Methods

### Precipitation and temperature datasets

We obtained daily gridded precipitation data from Asian Precipitation Highly Resolved Observational Data Integration Towards Evaluation of Water Resources (APHRODITE)^15^, which are available at 0.25° spatial resolution over the Asian Monsoon region (V1101R1) for the period of 1981–2007. The APHRODITE data (V1101R1) were developed using station based observations, which represent spatial variability and other rainfall characteristics well. For instance, APHRODITE data (here onwards: APHRO-Precipitation) represent orographic rainfall in the foothill of the Himalaya and the Western Ghats. The data are well quality controlled and checked for inconsistencies and errors^15^. Recently, Xie *et al.*^16^ used APHRO-Precipitation to investigate droughts in Pakistan while Duncan *et al.*^17^ used the dataset to analyse temporal trends in the Indian summer monsoon.

High-resolution near real-time daily and pentad (5 days total) precipitation data were obtained from the CHIRPS product (here onwards: CHIRPS-Precipitation)^11,18^, which are available at 0.25° and 0.05° spatial resolutions. CHIRPS-Precipitation is a combined product of monthly precipitation climatology (CHPClim), Thermal Infrared (TIR) satellite observations, and in situ precipitation observations from various national and regional meteorological departments^11,18^. The CHPClim is a long-term historical average rainfall accumulation, which is temporally disaggregated into 72 pentads (6-pentad per month) at a spatial resolution of 0.05° (ref. 19). The Thermal Infrared satellites, globally gridded Satellite (GriSat) (1981–2008), and Climate Prediction Center dataset (2000-present) are used in CHIRPS-Precipitation. The CHIRPS project generates a preliminary and a final product. The preliminary product is developed using CHPClim and TIR satellite data with a two-day lag. In situ observations stations are then blended with the preliminary data to produce the final product with a latency of about three weeks. More details about CHIRPS-Precipitation is available in Funk *et al.*^11^. Recently, Tote *et al.*^20^ used CHIRPS-Precipitation for drought and flood monitoring in Mozambique. Shukla *et al.*^21^ used CHIRPS-Precipitation for drought forecasting in East Africa. We used the final product of CHIRPS-Precipitation to develop pentad time-series from daily precipitation for the period 1981–2007, which is an overlapping period of APHRO-Precipitation.

Since a consistent long-term daily temperatures (maximum and minimum) dataset is unavailable for the South Asia, we obtained gridded daily maximum and minimum temperature data from the University of Princeton (here onwards: Princeton-Temperature^22^, http://hydrology.princeton.edu/data/pgf/0.25deg/daily/). Princeton-Temperature was obtained for the period 1970 to 2007 at 0.25°, which was developed using global observed datasets from the National Centers for Environmental Prediction (NCEP) and National Center for Atmospheric Research (NCAR) reanalysis data^22^. Princeton-Temperature was compared with the observed India Meteorological Data (IMD) and it was found that both the datasets are consistent with spatial and temporal variability and for correlations between precipitation and air temperature^23^. Recently, Princeton-Temperature was used to evaluate the changes in hydro-climatic variables over the Indian subcontinental basins^23^. Moreover, Princeton-Temperature and precipitation datasets have been widely used to assess global and regional drought characteristics^24–27^.

Since Princeton-Temperature is not available in near real-time, maximum and minimum 2-m air temperature data were obtained from the Global Ensemble Forecast System (GEFS) reforecast version 2 (here onwards: GEFS-Temperature)^28^ (https://www.esrl.noaa.gov/psd/forecasts/reforecast2/download.html) for the period of 1985 to present at 0.50°. GEFS-Temperature is generated on a daily basis at 0000UTC (at 3-hour interval) which contains ten perturbed forecast members and one control forecast member. GEFS-Temperature was regridded at 0.25° using the synergraphic mapping system (SYMAP) algorithm. The SYMAP algorithm uses temperature lapse rate using a high-resolution elevation, which is discussed in detail in Maurer *et al.*^29^. GEFS-Temperature has been used as a forcing in land surface models to evaluate drought severity, monitoring, and forecast^8,30–32^. We used a high resolution (2.5 arc minute, ≈5 km) maximum and minimum temperature climatology from the Worldclim version 2 (here onwards: WCLIM-Temperature)^33,34^ (http://worldclim.org/version2) to provide spatial variability in the regridded GEFS-Temperature at 0.05°. The WCLIM-Temperature monthly climatology is available for the period of 1970–2000, which was interpolated using the thin-plate smoothing spline algorithm implemented in the ANUSPLIN package^33,34^.

### Bias correction of precipitation and temperature

Model analysis and satellite data may have random errors and bias compared to observations due to inadequate sampling, algorithm imperfection, and lack of in-situ data^35^. We evaluated bias in CHIRPS-Precipitation and GEFS-Temperature against APHRO-Precipitation and Princeton-Temperature, respectively. There are several approaches available for bias correction^8,36–39^; we analysed two bias correction methods (linear scaling method^8,36^ and distribution mapping method^36,37^) to adjust the bias in CHIRPS-Precipitation and GEFS-Temperature (minimum and maximum) and selected the most efficient method of bias correction that can reduce bias effectively in near real-time.

CHIRPS-Precipitation was bias-corrected using the linear scaling and distribution mapping methods. In the linear scaling, bias correction of CHIRPS-Precipitation was performed in two steps as described in Shah and Mishra^8^; first, we applied the correction to extreme events (above 90 percent threshold value) and then, bias correction was applied to total annual precipitation. We, therefore, estimated two scaling factors for each of 12 calendar months: i) for extreme values (above 90 percent threshold value) and ii) for annual totals of precipitation for each grid cell. The monthly scaling factors for extreme events were determined by taking the ratio of the sum of the extreme precipitation data of APHRO-Precipitation and CHIRPS-Precipitation for the corresponding month. Then, pentad CHIRPS-Precipitation were corrected by multiplying the particular month’s scaling factors for each grid assuming that scaling factors remain constant for all six pentads within a month. After adjusting the extreme events of all months, we estimated monthly scaling factors for annual totals by taking the ratio of total precipitation of APHRO-Precipitation and CHIRPS-Precipitation for the corresponding month and corrected the pentad CHIRPS-Precipitation by multiplying the scaling factors.

In the distribution mapping method^36,40,41^, bias correction was performed by matching the cumulative distribution functions (CDFs) of datasets where a particular distribution (Gamma or Normal) was used to estimate the parameters. We used the Gamma distribution^42^ to fit pentad precipitation while empirical and normal distributions were used for temperature data.

To correct the precipitation data, we fitted the Gamma distribution to pentad precipitation to estimate the parameters for each month for APHRO-Precipitation and CHIRPS-Precipitation for each grid cell (0.25°). These monthly parameters for distributions were used for bias correction (to match the CDFS). We estimated the number of rainy pentads (>1 mm) in a given month for APHRO-Precipitation and CHIRPS-Precipitation for the training period. If CHIRPS-Precipitation has the number of wet pentads more than APHRO-Precipitation, we estimate the threshold value R_th_ using the equation (1). Using the threshold value and monthly parameters, we corrected pentad CHIRPS-Precipitation for the training period, and the method was evaluated for an independent data for the testing period. If the number of rainy pentads was more in the CHIRPS-Precipitation than APHRO-Precipitation for the training period, we used equation (2) to correct pentad CHIRPS-Precipitation otherwise used equation (3).

(1)Rth=cdfAPHRO−1(cdfCHIRPS(1))

(2)data_corri={cdfAPHRO−1(cdfCHIRPS(data_rawi))data_rawi>Rth0data_rawi≤Rth

(3)data_corri=cdfAPHRO−1(cdfCHIRPS(data_rawi))data_rawi>0

The bias correction of pentad CHIRPS-Precipitation involves two steps. Initially, we corrected pentad precipitation at 0.25° using the linear scaling and distribution mapping. Then final correction of pentad CHIRPS-Precipitation was performed at 0.05° using the most efficient bias-correction method among the two. We corrected CHIRPS-Precipitation against APHRO-Precipitation at 0.25° for the training (1981–2004) period, and the effectiveness of bias correction was evaluated for the testing (2005–2007) period. We disaggregated the APHRO-Precipitation from 0.25° to 0.05° spatial resolution^29^ to calculate the monthly scaling factors and parameters at 0.05°. We also evaluated the approach based on disaggregation of monthly scale factors and results were similar. These monthly parameters were used to correct CHIRPS-Precipitation at 0.05° using the same approach. The bias-corrected precipitation at 0.05° was aggregated to 0.25° and compared with the corrected CHIRPS-Precipitation at 0.25°, which was corrected with the APHRO-Precipitation at its native 0.25°. We found that both the bias corrected products resulted in a similar bias against the observed precipitation.

We also analysed the bias correction in 0.25° GEFS-Temperature (minimum and maximum temperature) against the observed Princeton-Temperature for training (1985–2004) and testing (2005–2007) periods using linear scaling method and distribution mapping (Q-Q mapping) method. In linear scaling method, we estimated monthly scale factors from both the temperature datasets for each month for each grid cell for the training period 1985–2004. The monthly scale factors were estimated by subtracting the mean monthly maximum or minimum GEFS-Temperature to Princeton-Temperature for the corresponding month. These monthly scale factors were subtracted from the raw pentad GEFS-Temperature for each month to obtain the corrected pentad GEFS-Temperature for each grid cell.

In the distribution mapping method^41^, we estimated empirical CDFs for each month for each grid from Princeton-Temperature and GEFS-Temperature for the training period. We corrected the GEFS-Temperature using CDFs mapping (Q-Q mapping)^41^ except extreme temperature events (less than 5 and more than 95% probability of exceedance). For extreme temperature events, normal distribution was employed. Further details on the linear scaling and distribution mapping method of bias correction in temperature data can be obtained from Shah and Mishra^8^ and Wood *et al.*^41^, respectively. Using the linear scaling and distribution mapping, we corrected the GEFS-Temperature for training and testing periods at the spatial resolution of 0.25° and selected the most efficient method for bias correction.

The adjusted maximum and minimum temperatures were regridded at the spatial resolution of 0.05° from 0.25° for the period 1970-present using high-resolution digital elevation model (for lapse rate) and the SYMAP algorithm as described in Maurer, *et al.*^29^. The regridded GEFS-Temperature data were again corrected using the WCLIM-Temperature climatology (1970–2000), which is available at 5 km using the linear scaling method. This correction was done to capture spatial variability of high-resolution WCLIM-Temperature data. Further details on the datasets used in this study are presented in Table 1.

### Drought indices

After the bias correction of CHIRPS-Precipitation and GEFS-Temperature at 0.05°, we estimated SPI and SPEI for drought assessment and monitoring. SPI and SPEI are commonly used dimensionless drought indices, which are estimated by fitting a probability distribution. SPI is used to measure precipitation deficit or surplus while SPEI also considers the effect of air temperature over multiple time scales. We used the Gamma distribution to estimate SPI. We used Hargreaves method^43^ to estimate potential evapotranspiration (PET), which is required for SPEI estimation. We used R SPEI package^44^ to determine SPEI using the log-logistic distribution. SPI and SPEI values indicate a wet condition for above zero and dry condition for below zero values. Normal (SPI/SPEI between −0.5 and 0.5), abnormal (SPI/SPEI between −0.5 and −0.8), moderate (SPI/SPEI between −0.8 and −1.2), severe (SPI/SPEI between −1.2 and −1.6), extreme (SPI/SPEI between −1.6 and −2.0), and exceptional (SPI/SPEI less than −2.0) category droughts were considered in our analysis^45^.

We used Normalized Difference Vegetation Index (NDVI) anomalies and the Drought Severity Index (DSI)^46^ to evaluate the drought estimates from CHIRPS-Precipitation and GEFS-Temperature in South Asia. We obtained MOD13A3 (Monthly L3 Global 1 Km NDVI) NDVI from the United States Geological Survey (USGS) MODIS Reprojection Tool Web Interface (MRTweb) (https://mrtweb.cr.usgs.gov/) from January 2002 to June 2016. 1 km NDVI was aggregated to 0.05° using the majority resample tool in ArcGIS. This monthly product is generated using 16-days 1-km NDVI product by employing a weighted temporal average value (if data are cloud free) and the maximum value (in the case of clouds)^47,48^. The MODIS vegetation indices are being used in Global Monitoring of vegetation health, modeling hydrologic process, drought monitoring, and global and regional climate change impacts assessment^47^. NDVI is widely used in drought assessment, drought and vegetation health monitoring^49–51^. Recently, Asoka and Mishra^52^ used NDVI and hydro-climatic variables (soil moisture and sea surface temperature) to predict vegetation anomalies for India.

## Data Record

High-resolution pentad precipitation, maximum and minimum temperature, SPI and SPEI datasets (Data Citation 1) are available from Figshare for the South Asia domain, which includes India, Pakistan, Nepal, Bhutan, Bangladesh, and Sri-Lanka. High-resolution datasets are also aggregated at district and sub-basin levels and freely available online through an open repository from 1981 to 2016, which will be extended to near real-time. Details on data format and columns can be obtained from a readme file provided at the above link.

## Technical Validation

**Figure 1:**
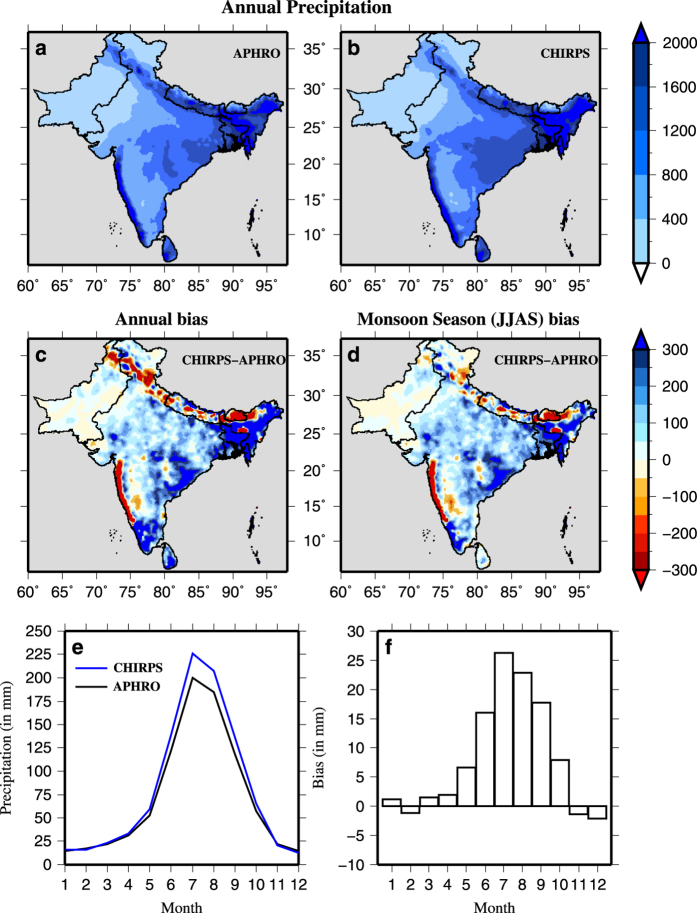
Annual and seasonal bias in CHIRPS precipitation in South Asia. (**a**,**b**) Mean annual precipitation (in mm) over the South Asia for the training period (1981–2004) from APHRODITE and CHIRPS. Bias in (**c**) mean annual precipitation and (**d**) during monsoon season (JJAS); (**e**) Mean monthly precipitation averaged over the South Asia and, (**f**) the bias in mean monthly precipitation for the training period.

We compared mean annual precipitation in CHIRPS-Precipitation against APHRO-Precipitation at 0.25° for the training period (1981–2004). We find that spatial variability of the APHRO-Precipitation is well represented in the CHIRPS-Precipitation (Fig. 1a,b). For instance, in both the datasets, regions with high (Western Ghats, North-East India, Bangladesh, Sri-Lanka, and South Bhutan) and low (Western India and parts of Pakistan) mean annual precipitation are well represented. During the training period, CHIRPS-Precipitation showed both dry and wet bias for mean annual and the monsoon season precipitation (Fig. 1c,d). CHIRPS-Precipitation shows underestimation in the Western Ghats and foothills of Himalayas while overestimation in North-East India, South India, and Sri Lanka. Relatively lower bias can be seen in CHIRPS-precipitation in Pakistan and the semi-arid regions of the western India (Fig. 1c,d). Moreover, in comparison to gauge based APHRO-Precipitation, CHIRPS-Precipitation overestimated rainfall during the monsoon season (Fig. 1e,f). We divided the entire South Asia into five regions and estimated bias in CHIRPS Precipitation for the training period (Supplementary Fig. S7). We notice random bias in CHIRPS-Precipitation, which can be associated with satellites, terrain, and spatial and temporal resolution^53^.

Corrected CHIRPS-Precipitation showed a considerably less bias for the both training and testing periods (Supplementary Fig. 1) at annual and seasonal scales (Supplementary Figs 5 and 8). The distribution mapping method showed better results in comparison to the linear scaling approach (Supplementary Table 1). The distribution mapping method has more advantages^36^ compared to the linear scaling as it performs efficiently in near real-time applications. Therefore, we selected the distribution mapping over the linear scaling method for bias correction. We estimated bias at annual (Supplementary Fig. 4) and seasonal time scales (Supplementary Fig. 2) and at the spatial resolutions of 0.25° and 0.05°. For the training period, Nash-Sutcliffe Efficiency (NSE) increased from 0.90 to 0.96 after the bias correction for monthly precipitation. Moreover, after the bias correction, median root-mean-square-error (RMSE) was reduced from 20 to 10 mm/month for monthly precipitation. The effectiveness of the bias correction approach was evaluated for 0.05° CHIRPS-Precipitation and substantial improvements were found (Supplementary Fig. 6).

Similar to CHIRPS-Precipitation, we estimated bias in GEFS-Temperature (maximum and minimum) against Princeton-Temperature at 0.25° using the linear scaling and distribution mapping methods (Supplementary Figs 9–14). GEFS-Temperature shows a high negative (cold) bias in the North-East India, Nepal, Bhutan, West Pakistan, and in the Southern Peninsula of India while positive (warm) bias in the central India and Bangladesh (Supplementary Figs 9 and 12). The bias in GEFS-Temperature may be due to GFS model input parameters as well as high terrain variability in the region especially in the Himalayas and Western Ghat. Moreover, GEFS uses the Climate Forecast System Reanalysis (CFSR) dataset as initial conditions^54^, which has a bias in precipitation and temperature over the Indian domain^55^. After the bias correction from both the methods, we compared bias in GEFS-Temperature (Supplementary Figs 9 and 12), and significantly less bias can be observed from both the methods in the GEFS-Temperature for the training and testing periods (Supplementary Figs 9 and 12; Supplementary Tables 2 and 3). However, some parts of North India show a negative bias in minimum and maximum temperatures, which can be attributed to sparse gauge network in the region^8,56^.Similar to precipitation, we estimated NSE and RMSE for both the methods for temperature (Supplementary Tables 2 and 3) and the distribution mapping performed better than the linear scaling.

**Figure 2:**
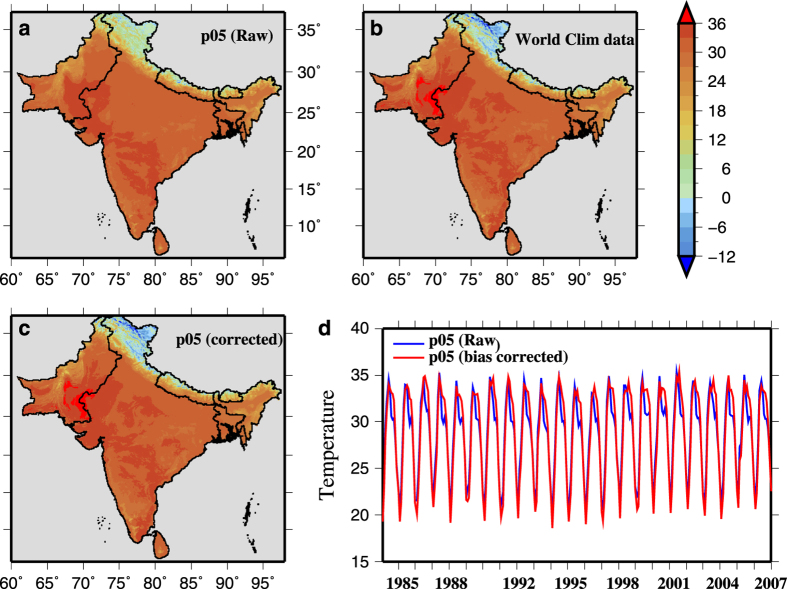
Bias-correction of maximum temperature from Global Ensemble Forecast System (GEFS). (**a**) Mean annual maximum temperature (°C) at 0.05° spatial resolution (regridded from corrected GEFS 0.25°) for the period 1985–2007, (**b**) mean annual maximum temperature from the Worldclim data, (**c**) corrected mean annual maximum temperature at 0.05° spatial resolution against the Worldclim data for the period 1985–2007, and (**d**) mean monthly maximum temperature averaged over the South Asia from raw and corrected maximum temperature.

We regridded the bias-corrected (using the distribution mapping) 0.25° GEFS-Temperature to 0.05° using a high-resolution digital elevation map (to provide lapse rate) and SYMAP algorithm as described in Maurer *et al.*^29^. After regridding the GEFS-Temperature, the dataset was further corrected using the monthly climatology of WCLIM-Temperature to capture finer scale spatial variability (Fig. 2 and Supplementary Fig. 15). We compared the spatial variability of unadjusted maximum and minimum temperatures data against the WCLIM-Temperature for the period 1970–2000 and found that unadjusted data showed less spatial variation in the Himalaya region, central India, and the Thar Desert in comparison to WCLIM-Temperature. Adjusted GEFS-Temperature after correction captures fine-scale spatial variability present in WCLIM-Temperature (Fig. 2c and Supplementary Fig. 15c). We compared domain averaged maximum and minimum temperatures of adjusted and unadjusted GEFS-Temperature and found a good agreement for the period 1985 to 2007 (Fig. 2d and Supplementary Fig. 15d). The final bias-corrected CHIRPS-Precipitation and GEFS-Temperature were used for the drought monitoring and assessment as well as for analysis of climate anomalies at 0.25° and 0.05°.

**Figure 3:**
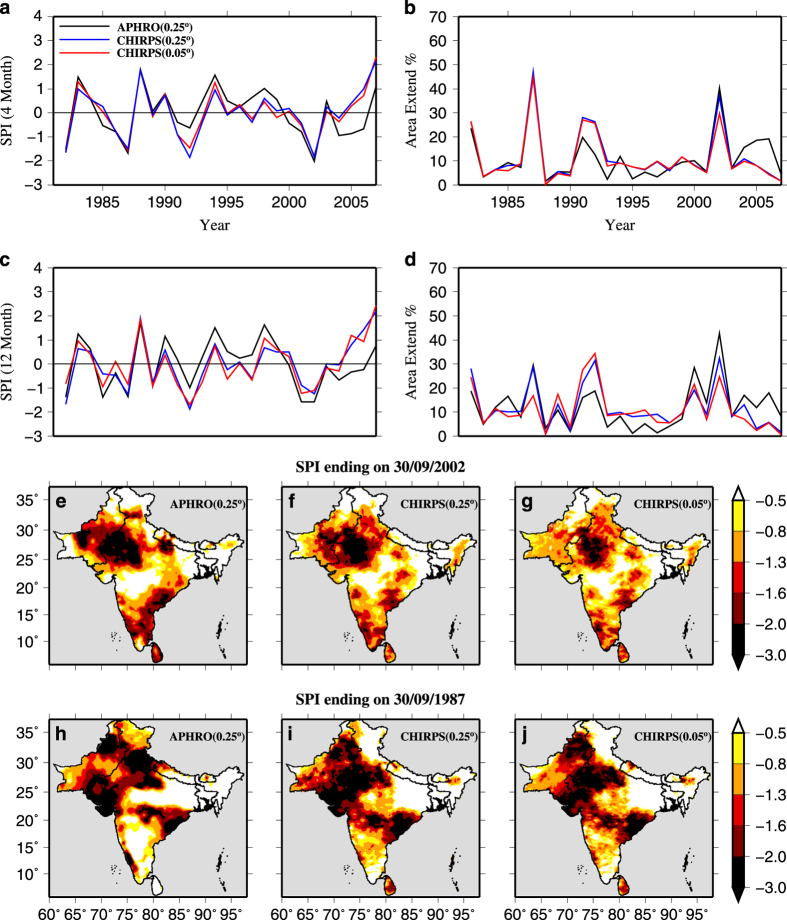
Drought estimates from APHRODITE and bias-corrected CHIRPS data using SPI. Meteorological drought estimated using 4-month SPI from APHRO-Precipitation (0.25°), bias-corrected CHIRPS-Precipitation (0.25°), and bias-corrected CHIRPS-Precipitation (0.05°) data. South Asia averaged (**a**) 4-month SPI at the end of September; (**b**) areal extent of drought in % (4-month SPI value less than −1.2); (**c**) 12- month SPI at the end of December; (**d**) areal extent of drought in % (12-month SPI value less than −1.2); (**e**–**g**) 4-month SPI at the end of September for the year 2002; and (**h**–**j**) 4-month SPI at the end of September for the year 1987.

We estimated SPI and areal extent (%) of drought using APHRO-Precipitation and bias corrected CHIRPS-Precipitation at 0.25° and 0.05° for the period of 1982–2007 over the South Asia (Fig. 3). The areal extent of drought was estimated for SPI threshold less than −1.2 (severe to exceptional droughts). We find that more than 25% of South Asia was under drought in 1982, 1987, 1992, 2002, and 2004 (Fig. 3 and Supplementary Fig. 16). SPI estimated from APHRO-Precipitation and bias corrected CHIRPS −Precipitation showed a similar temporal variation and areal extent of drought (Fig. 3a,d), however, with random bias. This variation in CHIRPS-Precipitation may be associated with the bias and other uncertainties due to the difference in the numbers of stations in monthly CHIRPS-v2.0 (ftp://ftp.chg.ucsb.edu/pub/org/chg/products/CHIRPS-2.0/diagnostics/stations-perMonth-byRegion/pngs/Southeast_Asia.station.count.CHIRPS-v2.0.png).

**Figure 4:**
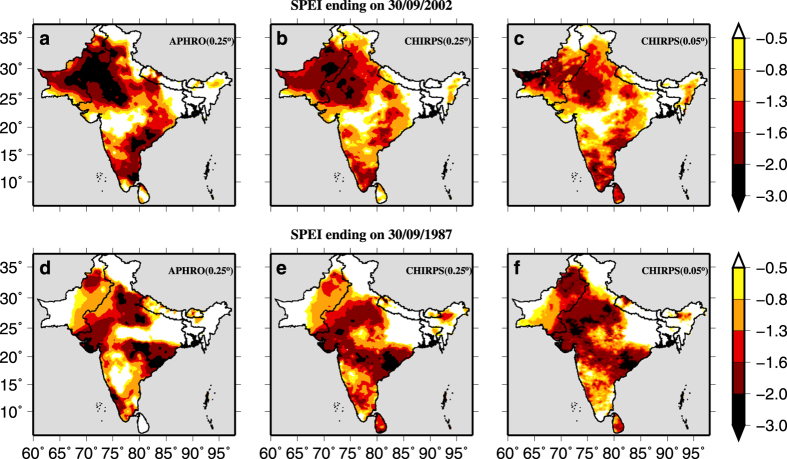
Drought estimates from APHRODITE and bias-corrected CHIRPS data using SPEI Drought estimated using SPEI (4-month) at the end of September of the year 2002 and 1987 using. (**a**,**d**) 0.25° APHRO-Precipitation, (**b**,**e**) 0.25° CHIRPS-Precipitation and (**c**,**f**) 0.05° CHIRPS-Precipitation.

We identified that the 1987 and 2002 were the two most severe drought years (areal extent more than 40%) during the entire period of 1981–2007. We compared drought severity and areal extents from CHIRPS-Precipitation (at 0.25° and 0.05°) and APHRO-Precipitation (at 0.25°) using 4-month SPI at the end of September, which represents accumulated precipitation during the monsoon season (Fig. 3e–j). Drought patterns obtained from CHIRPS-Precipitation were similar to APHRO-Precipitation in 2002. However, in 1987, 4-month SPI based on CHIRPS-Precipitation overestimated drought severity in the semi-arid western India. A similar comparison was done for drought estimates from SPEI, which accounts for the role of air temperature (Fig. 4). Results show that bias-corrected CHIRPS-Precipitation successfully captured areal extent and severity of droughts in 1987 and 2002 in the majority of regions in the South Asia.

**Figure 5:**
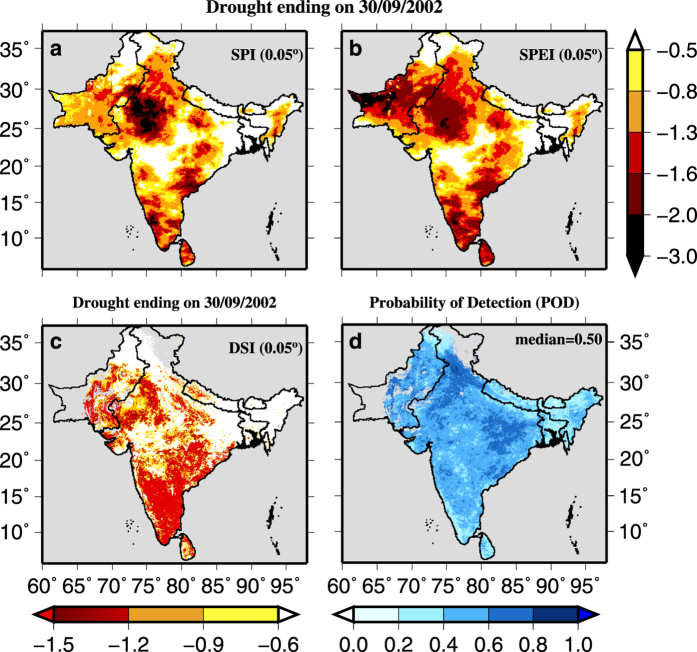
Evaluation of droughts from CHRIPS data against satellite driven DSI. Comparison of drought indices (SPI and SPEI) estimated from the high-resolution data with DSI. (**a**) 4- month SPI (**b**) 4- month SPEI, and (**c**) DSI at the end of September of the year 2002. (**d**) Probability of detection (4-month SPEI <−0.5) with reference to DSI (<−0.6) for the period 2000–2011.

To evaluate the role of air temperature on drought in South Asia, we compared 4-month SPI and SPEI at the end of the monsoon season of 2002 (Fig. 5a,b). We found that areal extents of droughts from 4-month SPI and SPEI estimated using the corrected CHIRPS-Precipitation and GEFS-Temperature (at 0.05°) were largely similar. However, differences in the severity can be noticed in the semi-arid western India and parts of Pakistan (Fig. 5a,b). We evaluated high-resolution SPI and SPEI estimated from the bias-corrected CHIRPS-Precipitation and GEFS-Temperature against satellite-based Drought Severity Index (DSI) at 0.05° for the 2002 monsoon season. We find that drought extent from SPI and SPEI obtained from the bias-corrected CHIRPS and GEFS data was well compared with that obtained from DSI (Fig. 5a–c). Differences in drought extent from DSI can be attributed to the presence of cloud cover and time-lag between precipitation and vegetation response. We estimated probability of detection (POD)^57,58^, which is a ratio of the total number of drought events when both DSI and 4-month SPEI showed the drought (DSI<−0.6 and SPEI<−0.5) to the total number of drought events when only DSI showed drought for the period of 2000–2011 (Fig. 5d). POD varies between 0 and 1, and lower values of POD represent the less correlation between DSI and SPEI. Based on the areal extent of drought and POD, we find a good agreement between DSI and SPEI demonstrating that the bias-corrected high-resolution data can successfully capture areal extent and severity of droughts in South Asia.

**Figure 6:**
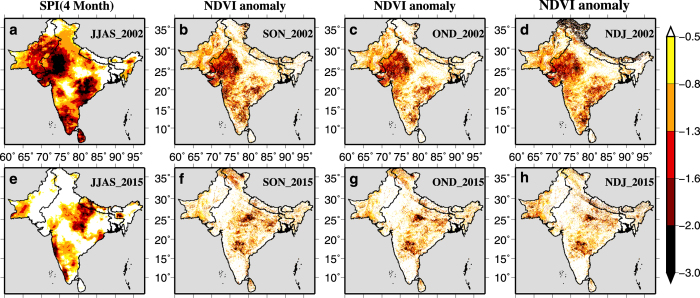
Linkage between drought and vegetation dynamics over the south Asia. Drought estimated using SPI and NDVI anomaly. (**a**,**e**) 4-month SPI at the end of September of the year 2002 and 2015. (**b**,**f**) September- October- November mean NDVI anomaly, (**c**,**g**) October -November-December mean NDVI anomaly, and (**d**,**h**) November- December- January mean NDVI anomaly for the year 2002 and 2015.

We estimated lag-correlation between 4-month SPI at the end of the monsoon season and 3-month averaged NDVI anomalies (Supplementary Fig. 17). We find that the drought during the monsoon season of 2002 was mainly centered in the western India and Pakistan, which resulted in vegetation stress in the following seasons (Fig. 6a–d). Similarly, the impacts of the monsoon season drought of 2015 can be noticed on vegetation during September to January period (Fig. 6e–h). These results highlight the utility of high-resolution drought monitoring in agriculture dominated South Asia.

**Figure 7:**
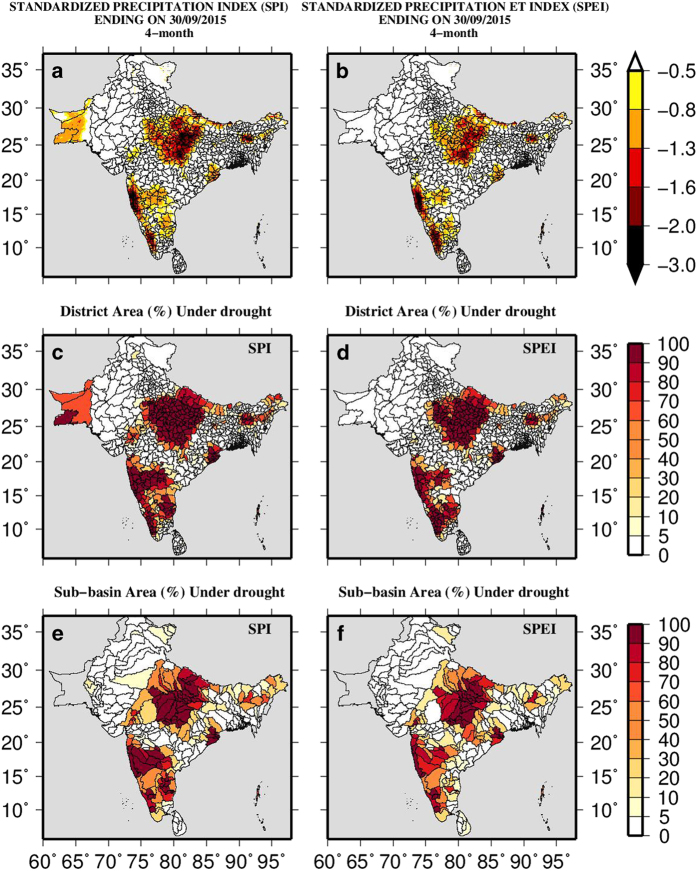
Intensity and areal extent of drought at sub-basin and district levels. District and sub-basin level drought monitoring using the SPI and SPEI over the South Asia. (**a**,**b**) 4- month SPI and SPEI; (**c**,**d**) district (%) area and (**e**,**f**) area (%) of sub-basins under drought at the end of September of the year 2015.

After a careful evaluation of bias-corrected datasets, we developed district and sub-basin level maps of drought severity and areal extent to assist in decision making (Fig. 7). 2015 was the 10th driest year in India and the top driest year in the Indo-Gangetic Plain during the record of 1906–2015 (ref. 2). Using the bias-corrected high-resolution data, we estimated the 4-month SPI and SPEI at the end of September 2015 to identify the areal extent of the monsoon season drought over the South Asia (Fig. 7a,b). 4-month SPI and SPEI show that a large area of India, Pakistan, and Nepal experienced severe and extreme drought in 2015. We also estimated areal extent (%) of drought at district and sub-basin levels over the South Asia (Fig. 7c–f), which can be used for decision and policy making.

**Figure 8:**
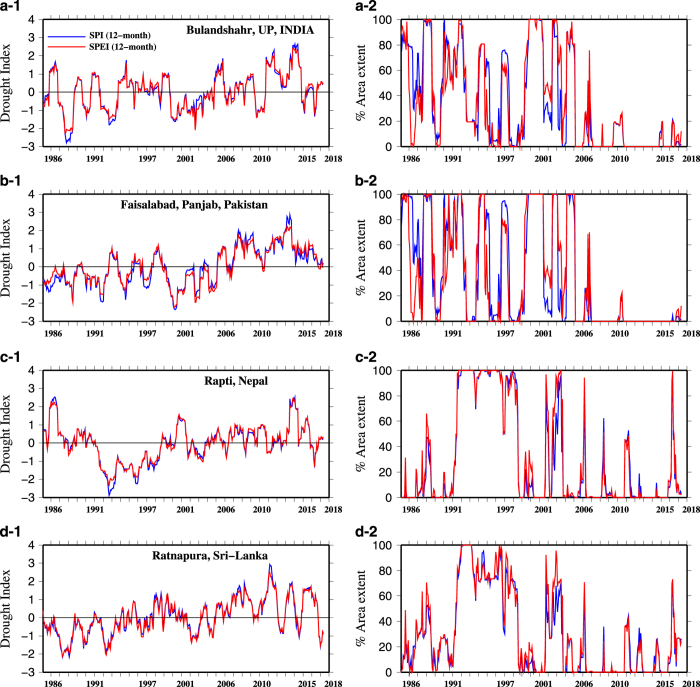
District level drought monitoring using the drought indices (SPI and SPEI) and areal extent (%) of drought. Drought condition for the district (**a**-1,2) Bulandshahr, U.P., India; (**b**-1,2) Faisalabad, Panjab, Pakistan; (**c**-1,2) Rapti, Nepal; and (**d**-1,2) Ratnapura, Sri-Lanka.

To further demonstrate the utility of the high-resolution dataset, we estimated 12-month SPI/SPEI and areal extent of droughts (based on SPI or SPEI <−1.2) for four districts (Fig. 8). These four districts are; District 1 (Bulandshahr, U.P., India), District 2 (Faisalabad, Punjab, Pakistan), District 3 (Rapti, Nepal), and District 4 (Ratnapura, Sri-Lanka). For instance, based on 12-month SPI and SPEI, Bulandshahr and Rapti districts show about 40–50 % area under the drought at the end of December 2015. On the other hand, Faisalabad and Ratnapura districts show no drought condition at the end of December 2015. District and sub-basin level administrators and water managers can use such information for near real-time monitoring and decision making. The high-resolution bias corrected data can also be used for monitoring and assessment of hydroclimatic extremes such as extreme precipitation, heat and cold waves (Supplementary Fig. 18). We evaluated November- December precipitation pentad anomaly to get the information about the 2015 Chennai flood. Extreme precipitation pentads were recorded in CHIRPS data during the November-December 2015. We also evaluated May-2015 and January-2016 GEFS-Temperature pentad to access the information of heat and cold waves respectively. These results further demonstrate the use of the dataset in one of the most populated regions of the world.

## Usage Notes

Drought events have increased in South Asia during the recent decades. A high-resolution near real-time drought monitoring in South Asia can be used for decision-making and to redefine policies in the areas of water management and agriculture. We bias-corrected precipitation and temperature datasets from CHIRPS and GEFS at 0.25° and 0.05° using the distribution mapping. The primary requirement of real-time drought monitoring is to have long-term data extended till near real-time. The high-resolution dataset can be used for drought monitoring and assessment at the district and sub-basin level in near real-time. Moreover, the dataset can also be used to monitor and assess hydroclimatic anomalies in one of the most populated and agriculture intensive regions of the world. The bias corrected pentad precipitation from CHIRPS and maximum and minimum temperatures from GEFS are available at 0.05° from the 1981-till present. Using the high-resolution bias corrected data, drought indices (SPI/SPEI) were estimated and evaluated against satellite-based drought products. The validation provides the confidence that the dataset can capture areal extent and severity of drought at district and sub-basin levels in South Asia.

While we have evaluated the high resolution dataset using satellite driven vegetation and drought indices, there are certain assumptions and limitations. For instance, we assumed that APHRO-Precipitation represents orographic and spatial variability after disaggregation from 0.25° to 0.05. Moreover, APHRO-Precipitation may have bias compared to other observed (e.g., IMD) data^59^ in some regions due to the less number of gauge stations, which might have affected the bias-correction of the CHIRPS data. Despite these limitations, the performance of bias-corrected CHIRPS data was satisfactory at both 0.25 and 0.05 resolutions. Similarly, for air temperature, we assumed that GEFS-Temperature captures the effect of elevation (lapse rate) and spatial variability after disaggregation to the higher resolution. GEFS-Temperature was bias corrected using the Princeton and WCLIM-Temperature, which might also have bias due to sparse gauge stations in some regions of the South Asia. Overall, areal extent of droughts was monitored well using the bias-corrected CHIRPS-Precipitation and GEFS-Temperature. However, like any other gridded dataset, there may be limitations and uncertainties related to bias correction and interpolation methods. Random errors may be generated or modified during the bias correction and interpolation of the precipitation and temperature datasets^29,36,60–63^. The dataset can be improved further based on the increased availability of observational stations in future.

High resolution (0.05°) datasets are aggregated at district and sub-basin levels and uploaded in Ascii (text) format from 1981 to present for every district and sub-basin in the South Asia. The data include precipitation, maximum and minimum temperatures for each pentad from 1981 to 2016. We also provide SPI/SPEI values for each district/sub-basin for each pentad considering 1–48 months duration to provide information on short to long-term droughts. The datasets can be easily used for drought analysis in any district and sub-basin in South Asia. Users can download data, and select country (India, Pakistan, Bangladesh, Bhutan, Nepal, and Sri Lanka), state, district, and sub-basin. The analysis can be performed for retrospective and near real-time monitoring and assessment of hydroclimatic extremes (drought, flood, and heat waves). The dataset will be updated on a regular basis to make it near-real time. For gridded data (0.05 degree), interested users can directly contact to the corresponding author. More instructions on data format and details can be obtained from a readme file provided in the data link.

## Additional information

**How to cite this article:** Aadhar, S. & Mishra, V. High-resolution near real-time drought monitoring in South Asia. *Sci. Data* **4**:170145 doi: 10.1038/sdata.2017.145 (2017).

**Publisher’s note:** Springer Nature remains neutral with regard to jurisdictional claims in published maps and institutional affiliations.

## Supplementary Material



Supplementary Information

## Figures and Tables

**Table 1:** Details of the datasets used in the study.

**Data**	**Sources**	**Spatio-temporal resolution**	**Availability**
APHRO-Precipitation	http://www.chikyu.ac.jp/precip/english/	0.25°, Daily	1951–2007
CHIRPS-Precipitation	http://chg.geog.ucsb.edu/	0.05° and 0.25°, Daily and Pentad	1981-present
Princeton-Temperature	http://hydrology.princeton.edu/data/pgf/0.25deg/daily/	0.25°, Daily	1948–2007
GEFS-Temperature	https://www.esrl.noaa.gov/psd/forecasts/reforecast2/	0.5°, 3-hourly	1984-present
WCLIM-Temperature	http://worldclim.org/version2	2.5 arc minutes (0.05°), Average monthly	1970–2000
Drought Severity Index	http://www.ntsg.umt.edu/project/modis/dsi.php	0.05°, 8 days	2000–2011
NDVI data	https://lpdaac.usgs.gov/dataset_discovery/modis/modis_products_table/myd13a3	0.01°, Monthly	2000-present
