# Supplementary material for: High-resolution near real-time drought monitoring in South Asia
Source: Sci Data. 2017 Oct 3;4:170145. doi: 10.1038/sdata.2017.145 (PMC5625554; doi:10.1038/sdata.2017.145)
Supplement: Supplementary Information [file sdata2017145-s2.docx]

### Supplemental Information

### Title

**High-resolution near real-time drought monitoring in South Asia**

### Authors

Saran Aadhar^1^, Vimal Mishra^1^

**Affiliations**

1. Civil Engineering, Indian Institute of Technology (IIT), Gandhinagar, India.

Corresponding Author: Vimal Mishra ([vmishra@iitgn.ac.in](mailto:vmishra@iitgn.ac.in))

**Contents of this file**

Figures S1 to S18

Tables S1 to S3


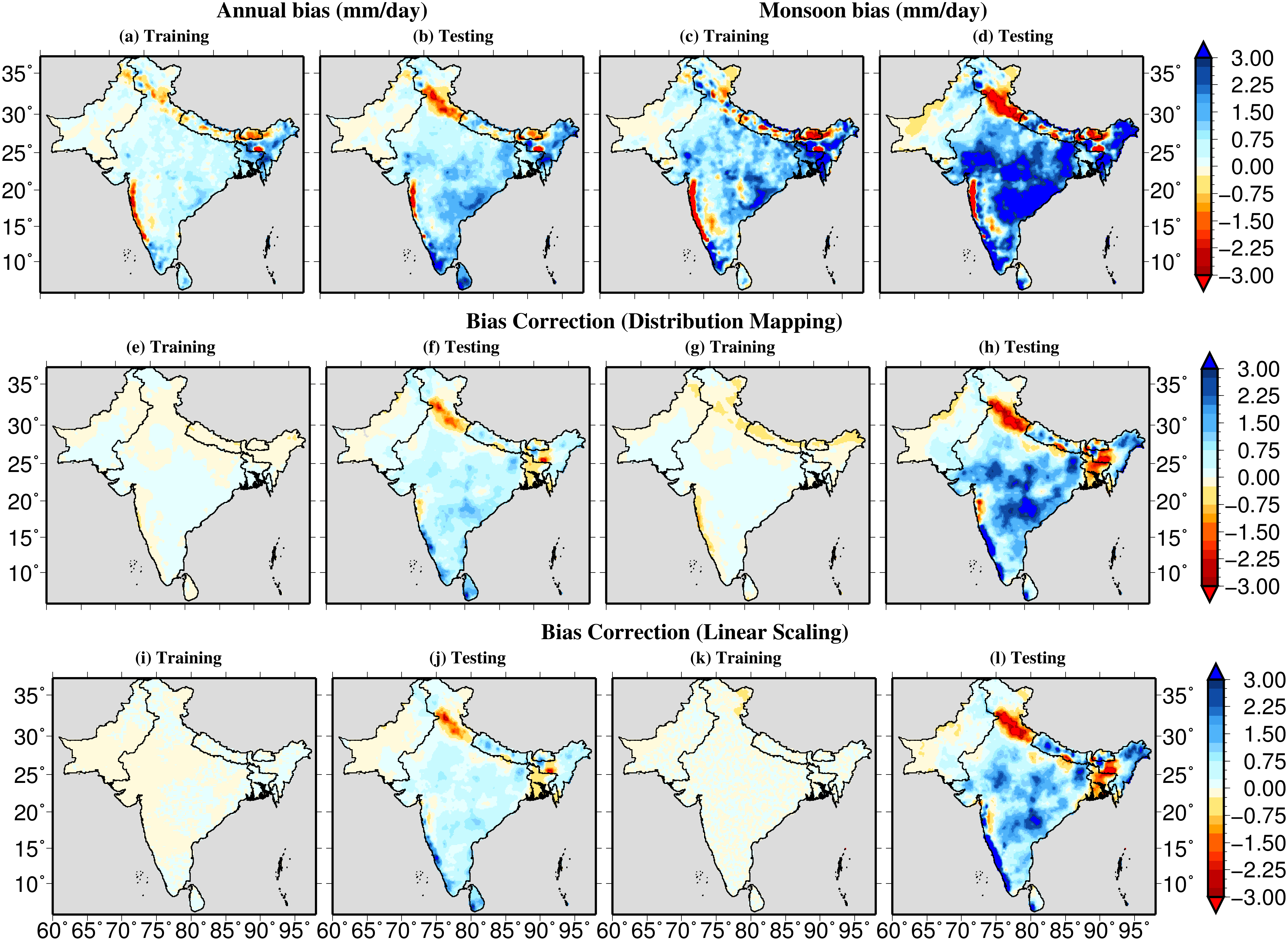


Figure S1. Comparison of bias against APHRO-Precipitation data in the raw (a-d) and corrected CHIRPS-Precipitation using distribution (Gamma) mapping (e-h) and linear scaling (i-l) method. Bias (mm/day) in mean annual precipitation for the (a, e, i) training period (1981-2004) and (b, f, j) testing period (2005-2007). Bias (mm/day) in the monsoon season (JJAS) precipitation for the (c, g, k) training period and (d, h, l) testing period.


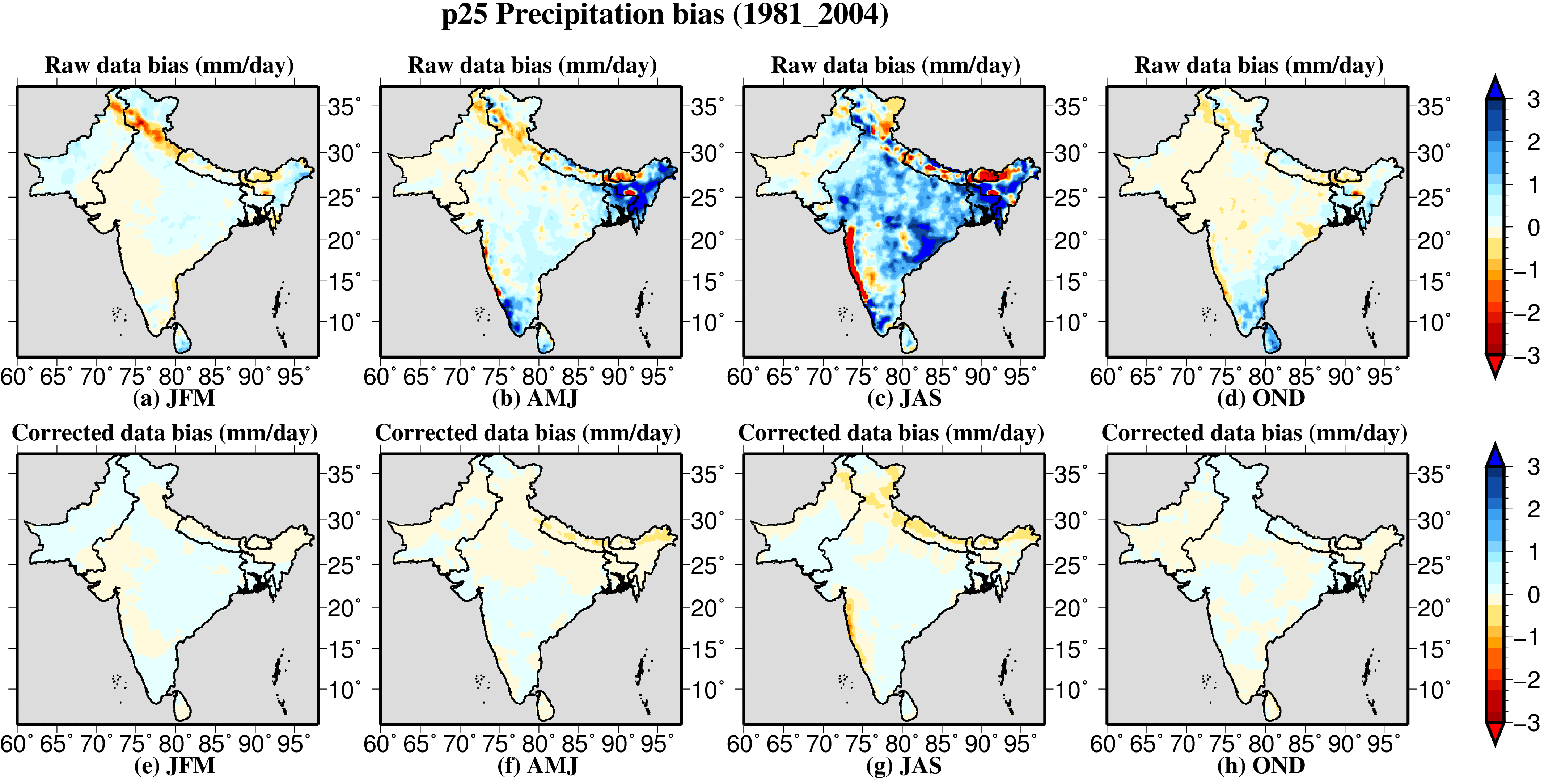


Figure S2. A comparison of bias in raw CHIRPS (0.25°) and bias-corrected CHIRPS (0.25°) data (using distribution mapping method) against APHRODITE (0.25°) data for the training period (1981-2004). The bias in (a) raw and (c) corrected CHIRPS data for JFM (January, February, March); (b, f) for AMJ; (c, g) for JAS; (d, h) for OND at the spatial resolution of 0.25° using distribution mapping method.


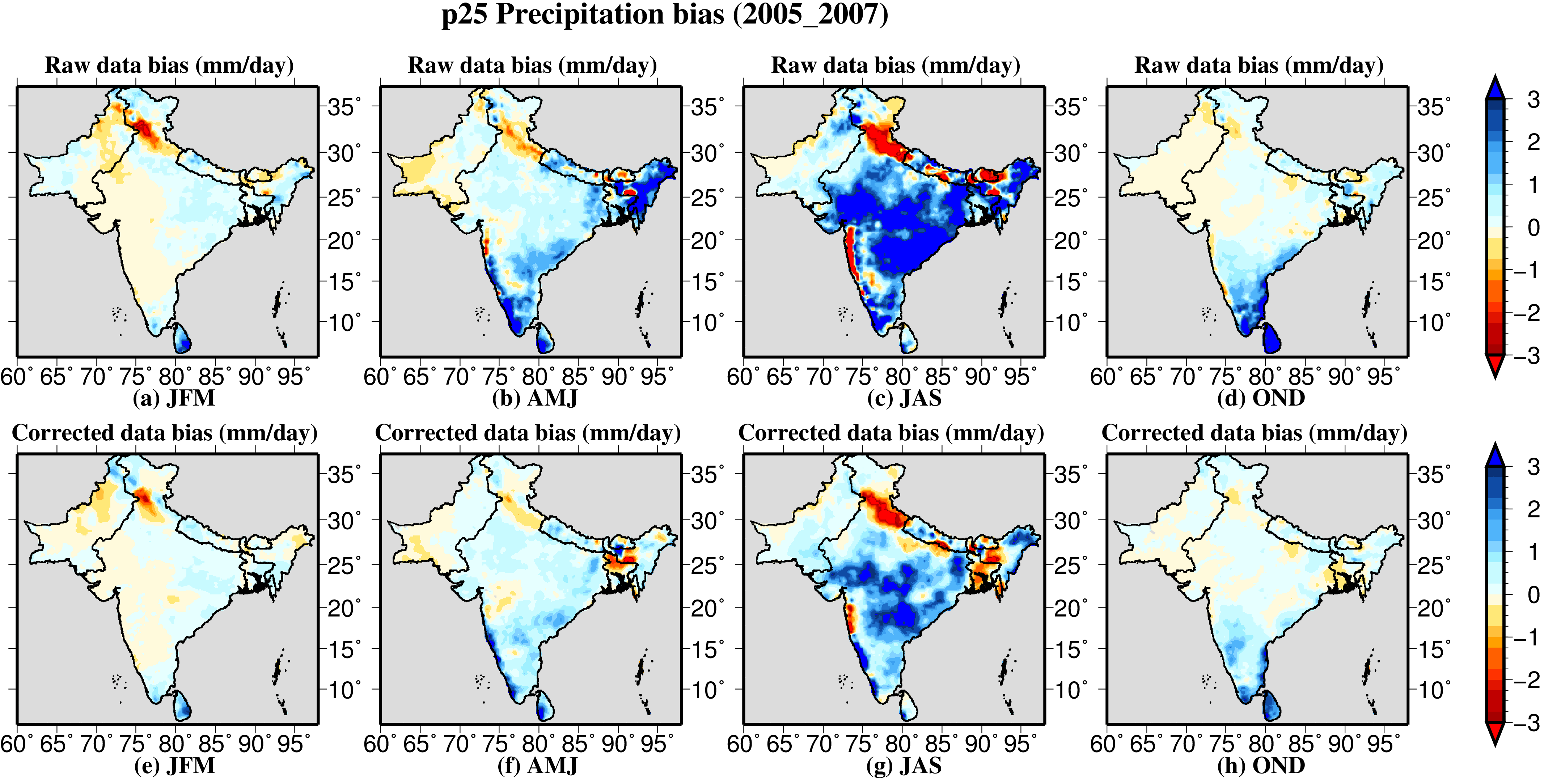


Figure S3. As in Figure S1, but for the testing period (2005-2007).


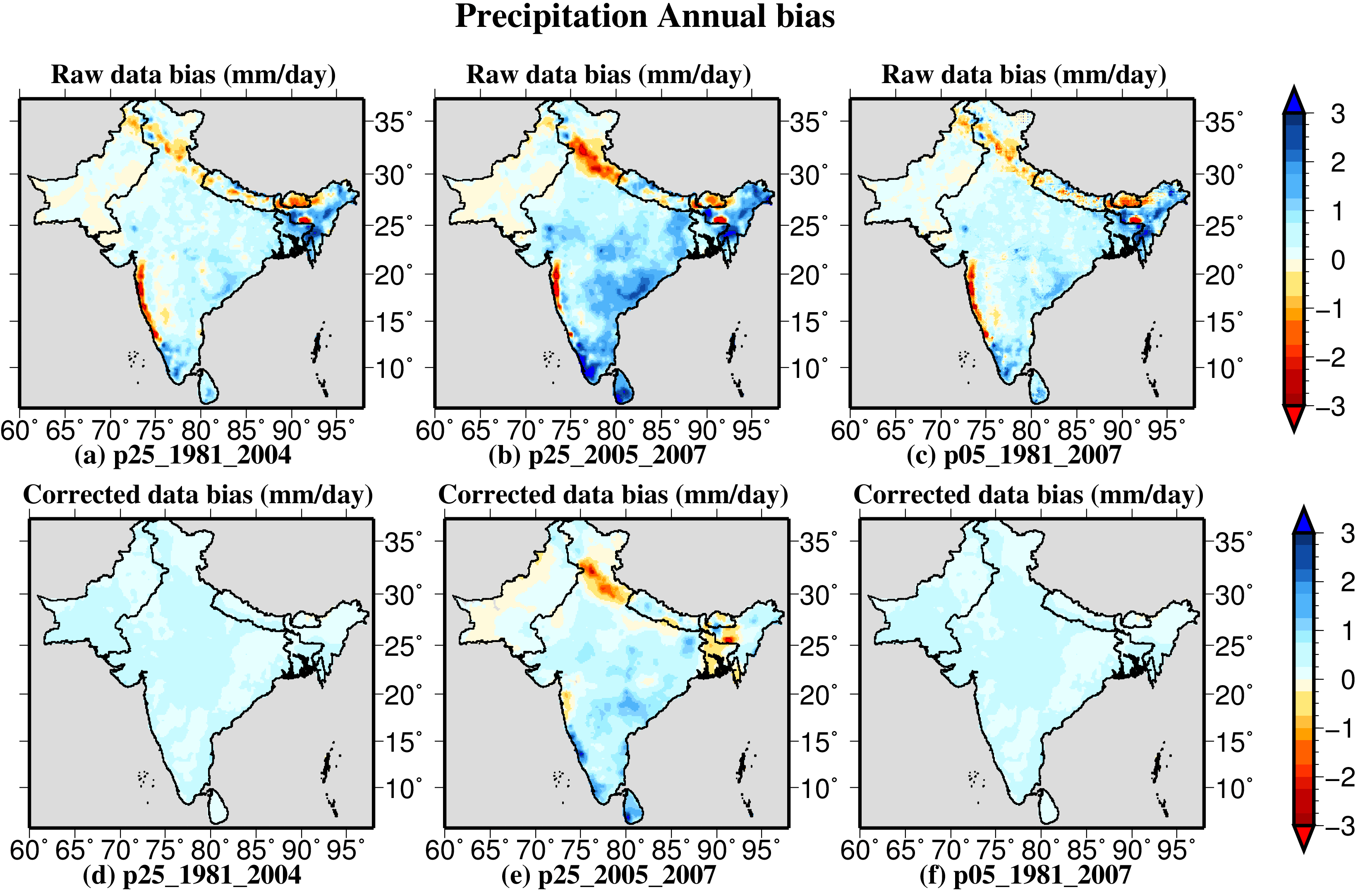


Figure S4. The annual bias in (a) raw and (d) corrected CHIRPS-Precipitation (using distribution mapping method) for the training period (1981-2004), (b) and (e) for the testing period (2005-2007) at 0.25˚ spatial resolution. The bias in (c) raw and (f) bias-corrected for the period of 1981-2007 at the spatial resolution of 0.05˚.


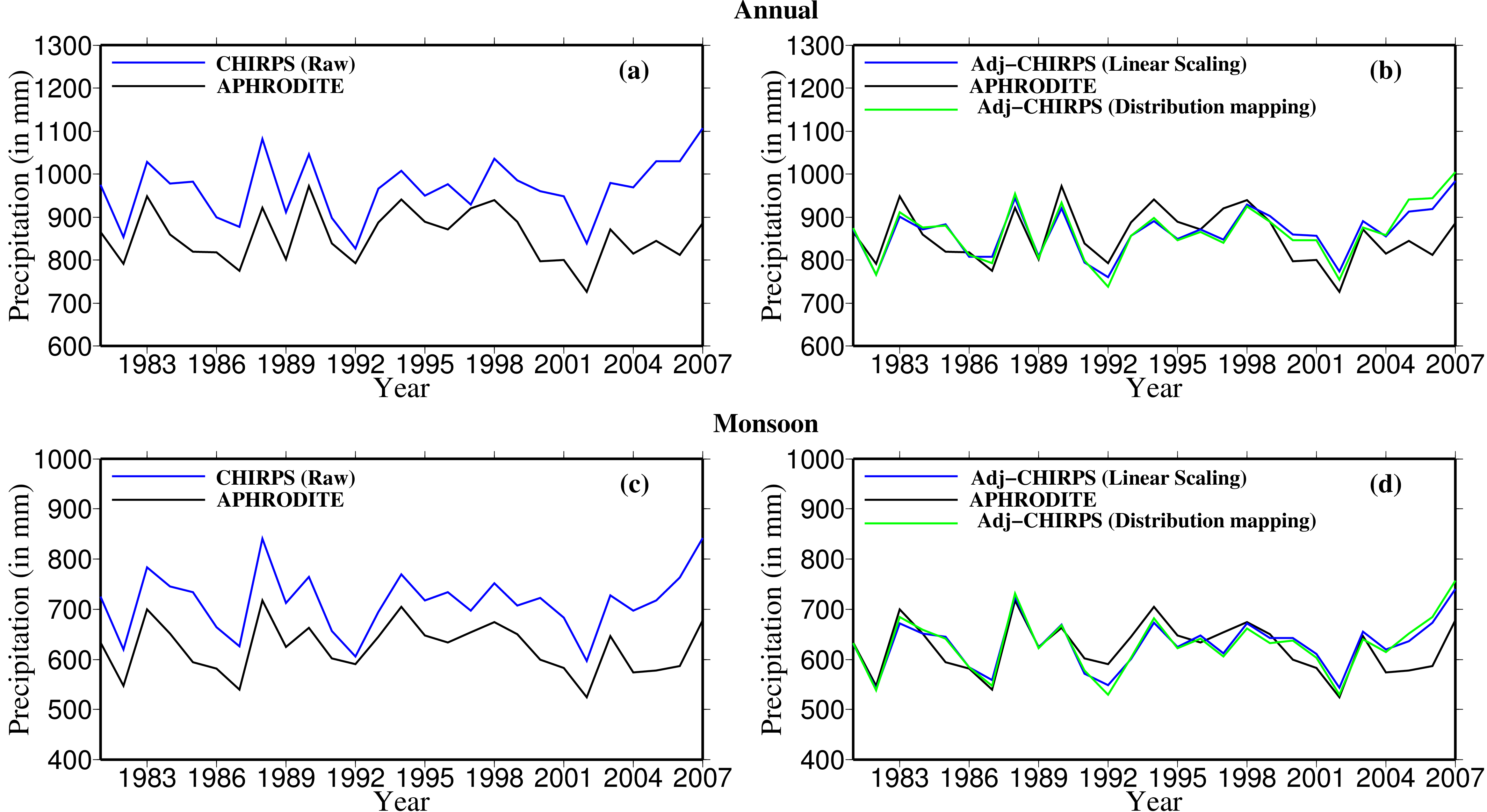


Figure S5. Effectiveness of the bias correction of CHIRPS data. Comparison of APHRO-Precipitation with raw and bias-corrected CHIRPS-Precipitation using the distribution mapping and linear scaling. (a,b) mean annual and (c,d) monsoon (JJAS) season precipitation averaged over the South Asia.


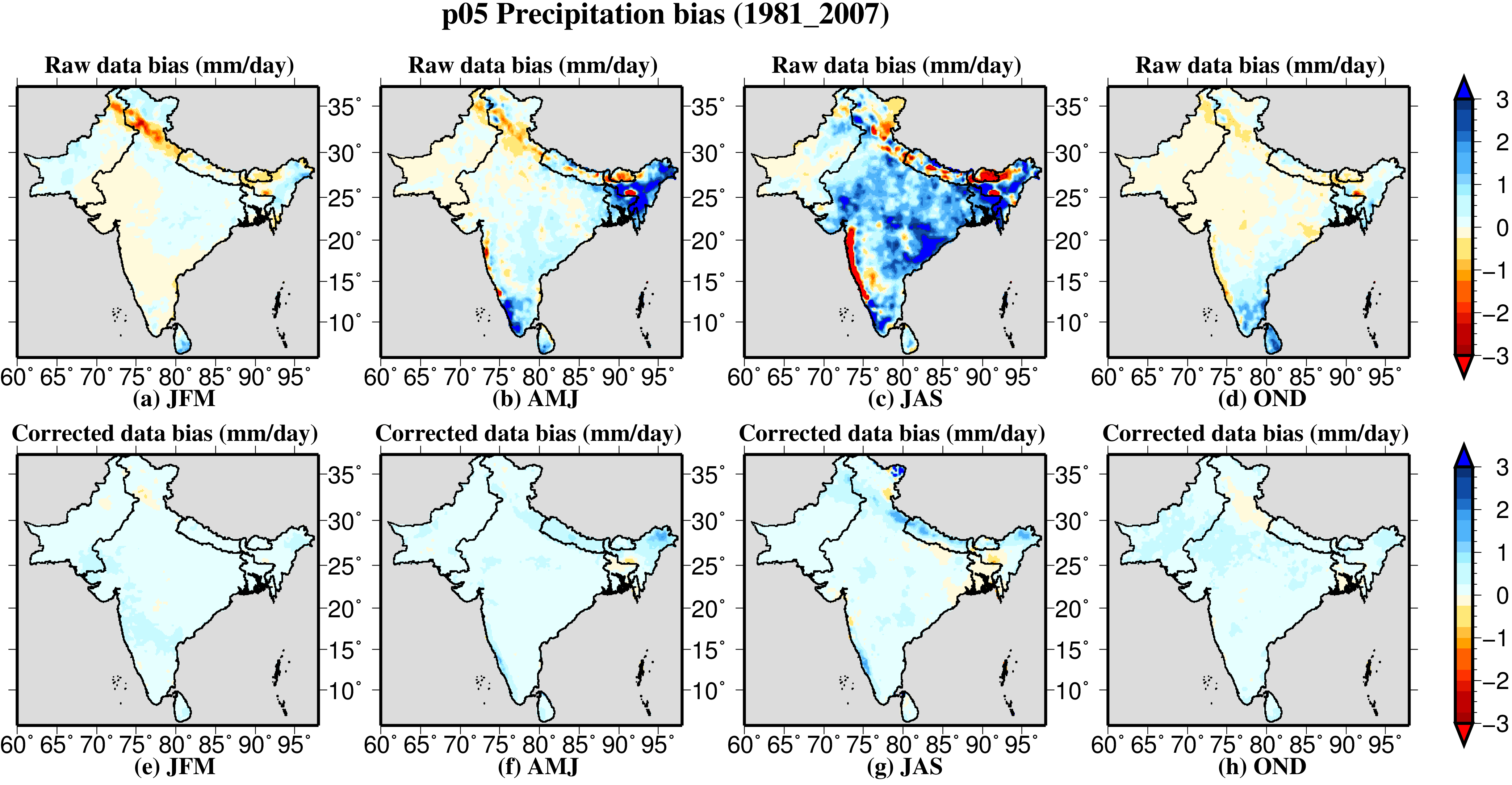
Figure S6. A comparison of bias in raw CHIRPS (0.05°) and corrected CHIRPS (0.05°) data (using distribution mapping method) against APHRODITE (0.25°) precipitation data for the period (1981-2007). The bias in (a) raw and (e) corrected CHIRPS data for JFM (Jan., Feb., March); (b, f) for AMJ; (c, g) for JAS; (d, h) for OND.


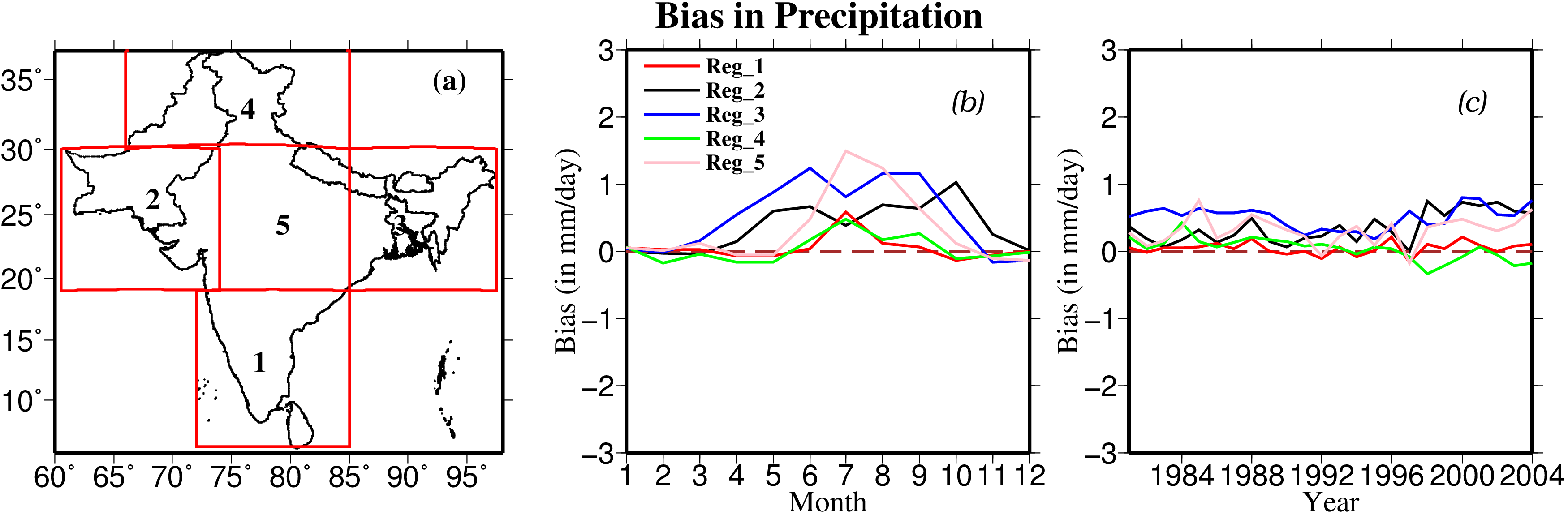


Figure S7 Region-wise bias in (b) mean monthly (Seasonal cycle) and (c) annual raw CHIRPS precipitation for the period 1981-2004.


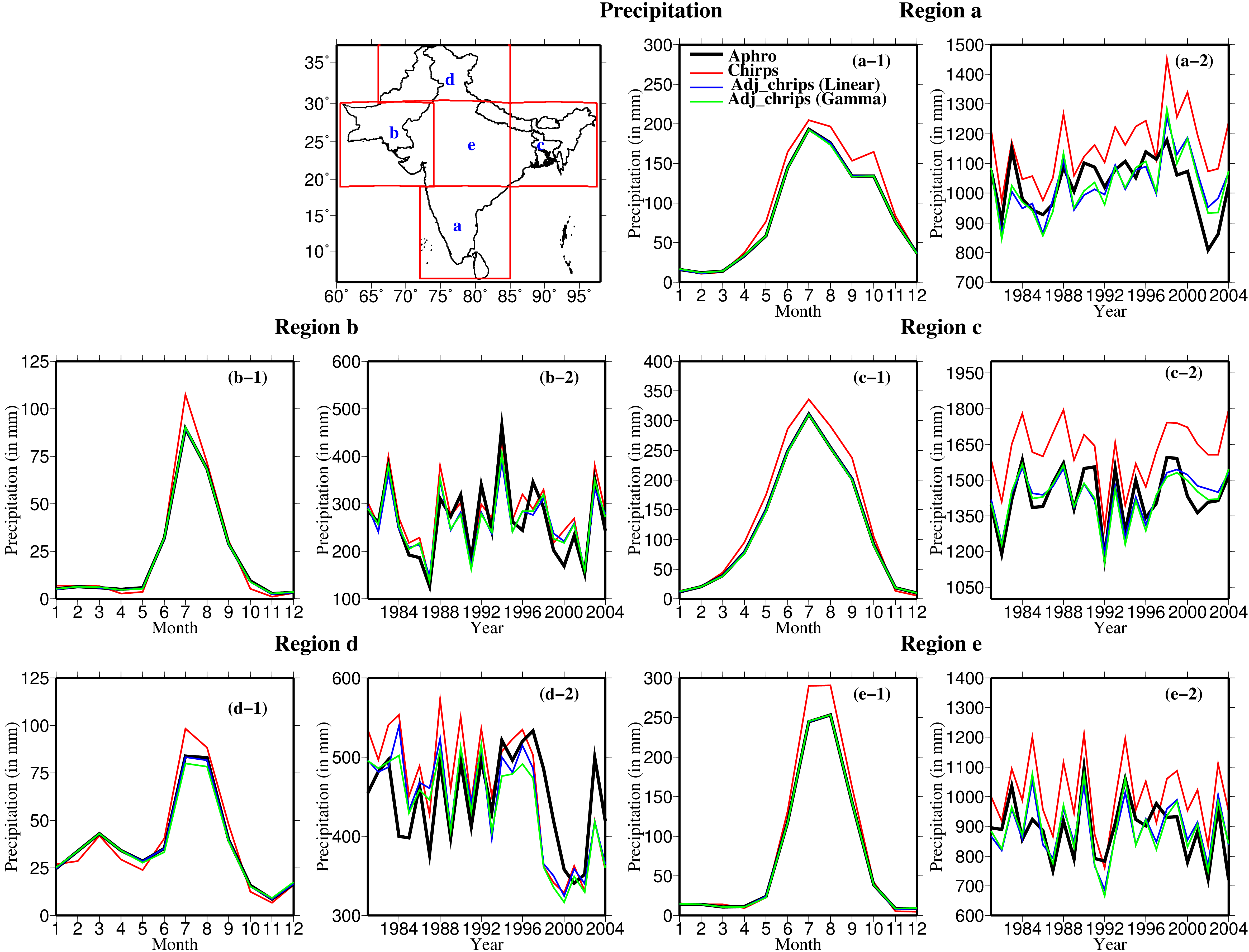


Figure S8 Region-wise averaged monthly (seasonal cycle) and annual precipitation (APHRO, CHIRPS (Raw), Corrected CHIRPS (linear), and Corrected CHIRPS (Gamma) Precipitation) for the period 1981-2004.


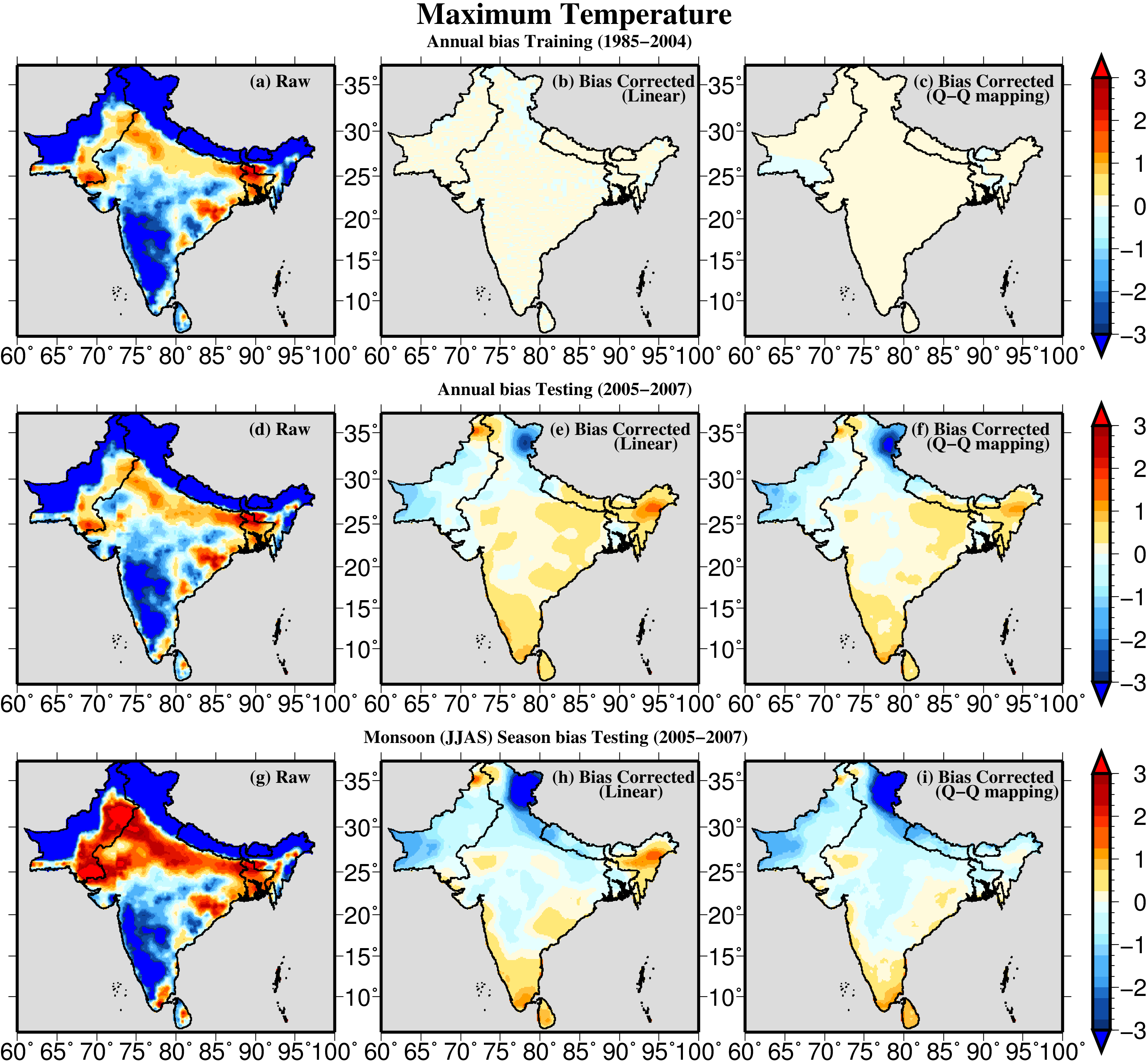


Figure S9. Comparison of mean maximum temperature (°C) in raw GEFS and bias-corrected GEFS temperature data against Princeton-Temperature data. The bias in (a) raw, (b) bias-corrected GEFS data using linear scaling method, and (c) bias- corrected GEFS using (Q-Q mapping) for the training period (1985–2004), (d- f) bias in annual mean maximum temperature for the testing period (2005–2007), and (g- i) bias in the monsoon season during the testing period.


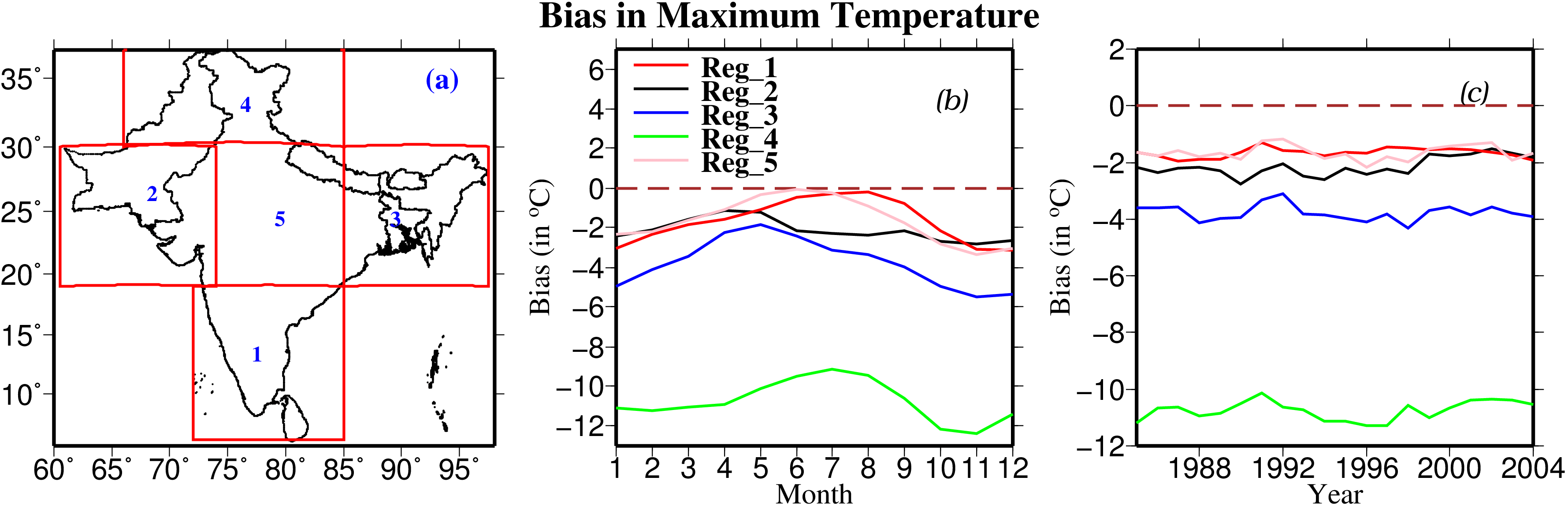


Figure S10 Region-wise bias in (b) mean monthly and (c) annual maximum GEFS (Raw) temperature for the period 1985-2004.


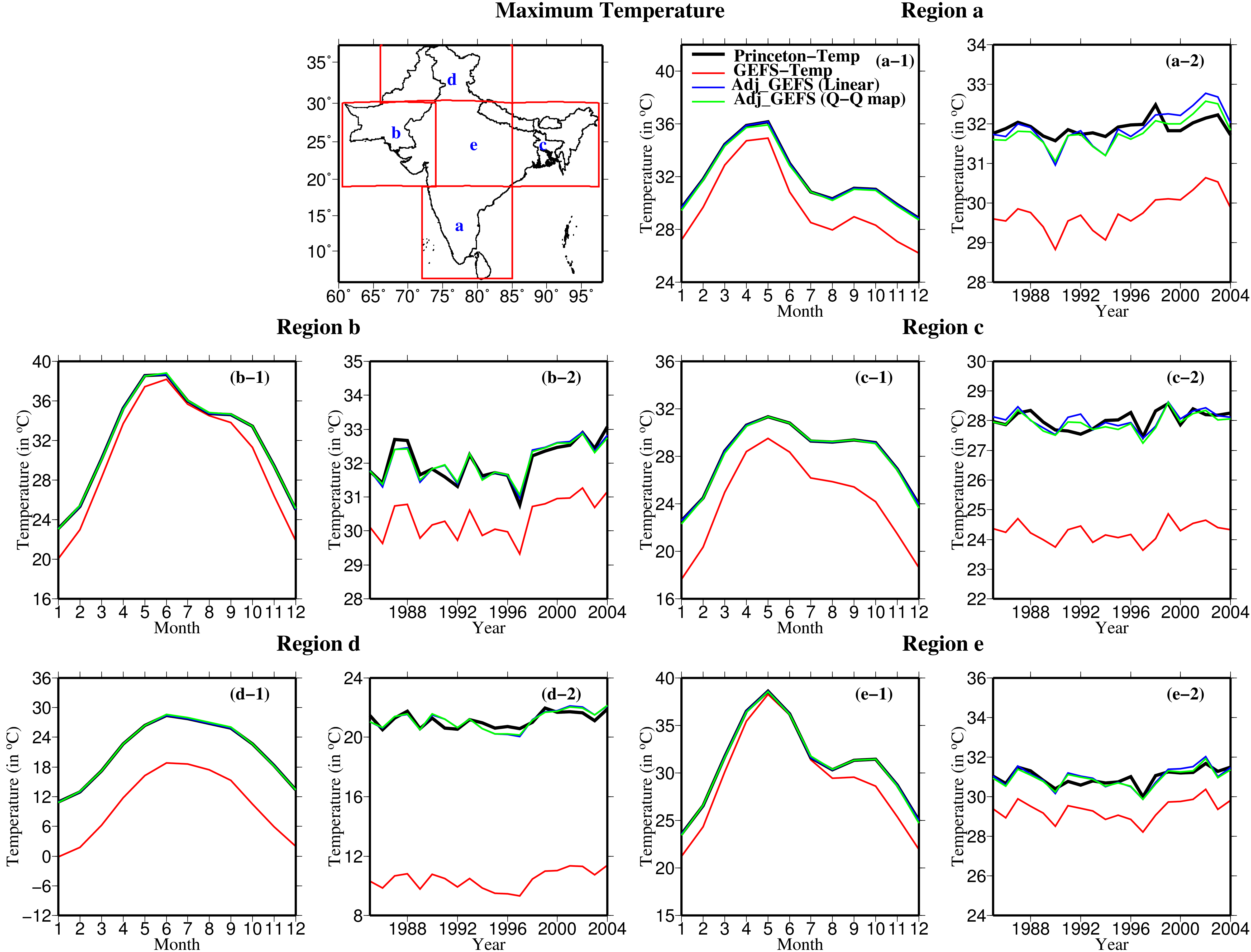


Figure S11 Region-wise averaged monthly (Seasonal cycle) and annual maximum temperature (Princeton, GEFS (Raw), Corrected GEFS (linear), and Corrected GEFS (Q-Q mapping)) for the period 1981-2004.


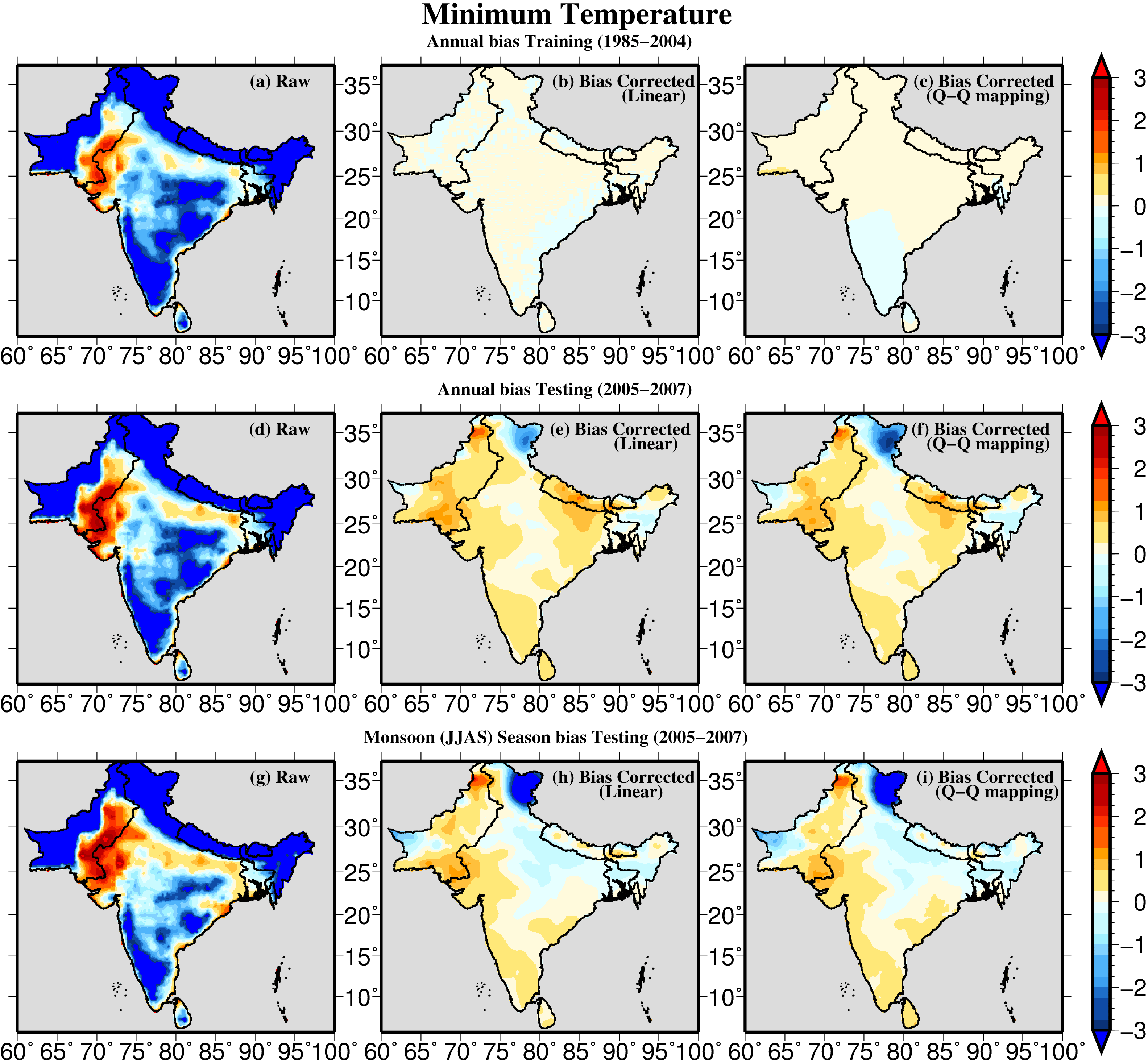


Figure S12 As in Figure S9, but for minimum temperature.


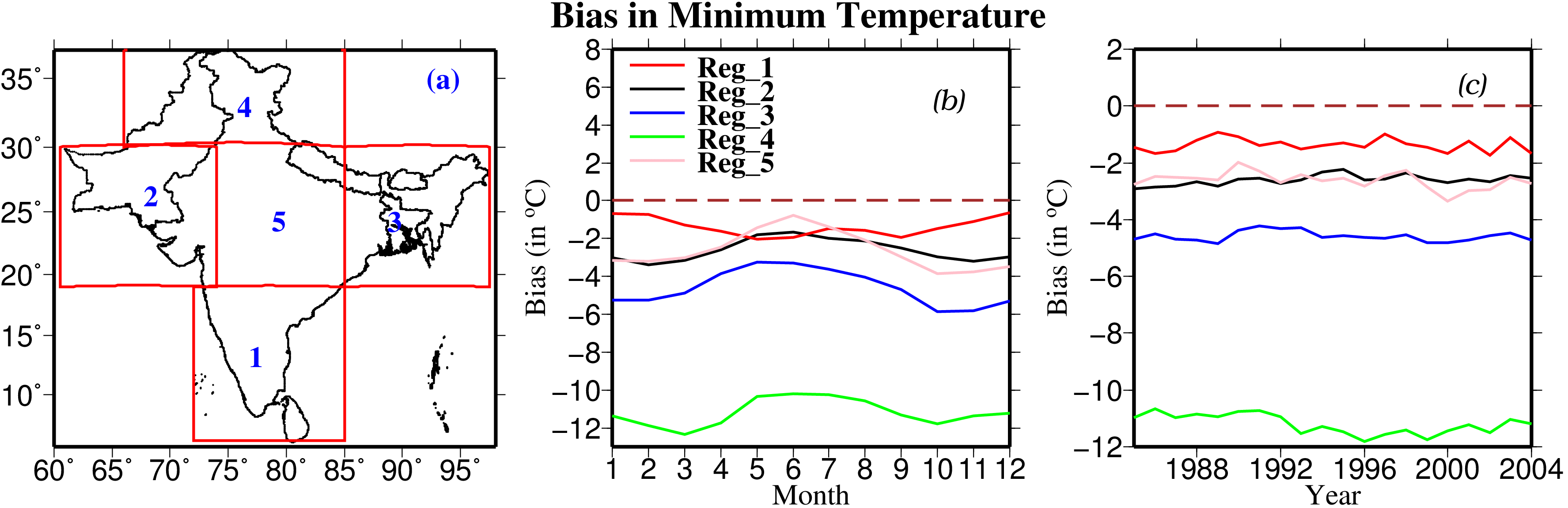


Figure S13 As in Figure S10, but for minimum temperature.


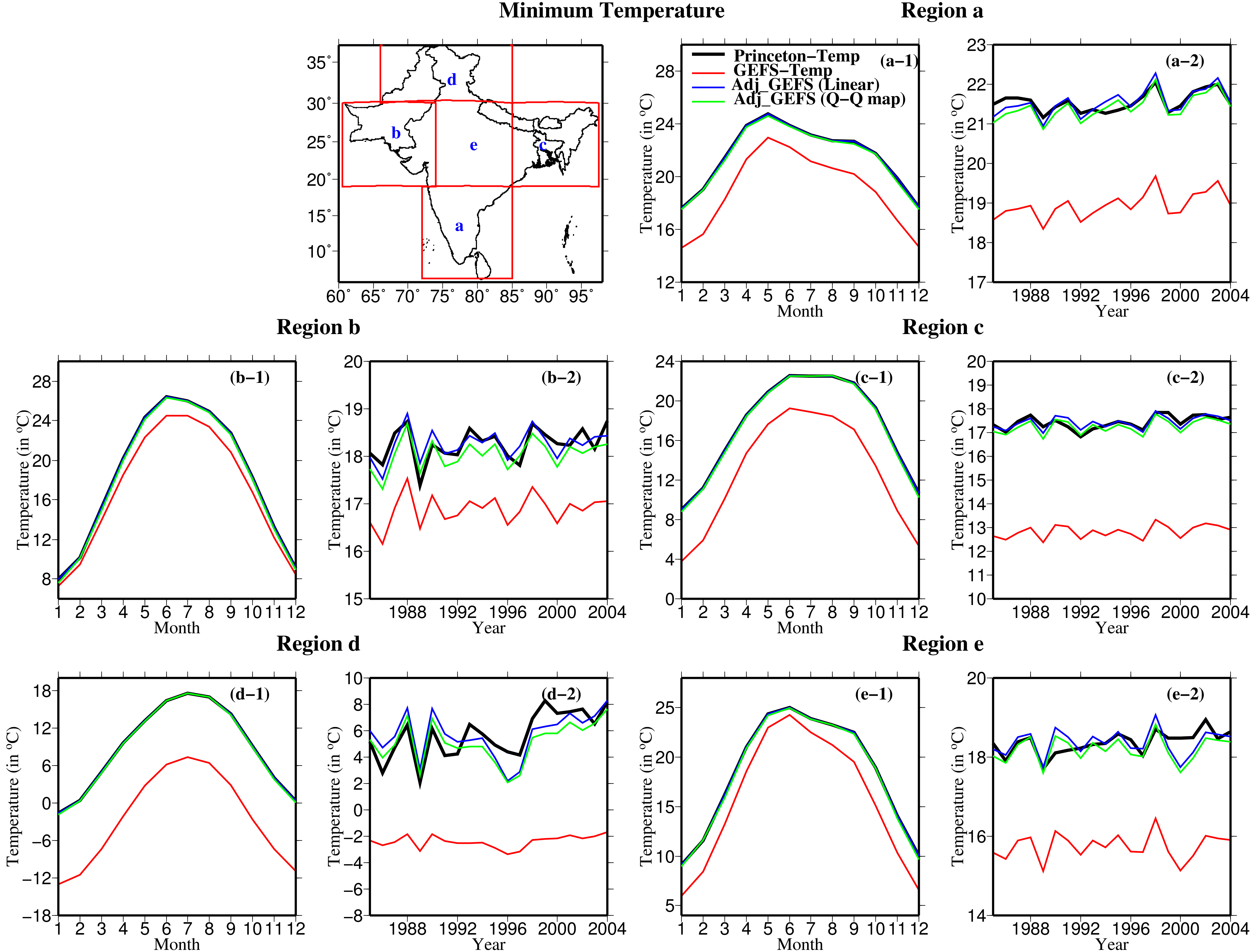


Figure S14 As in Figure S11, but for minimum temperature.


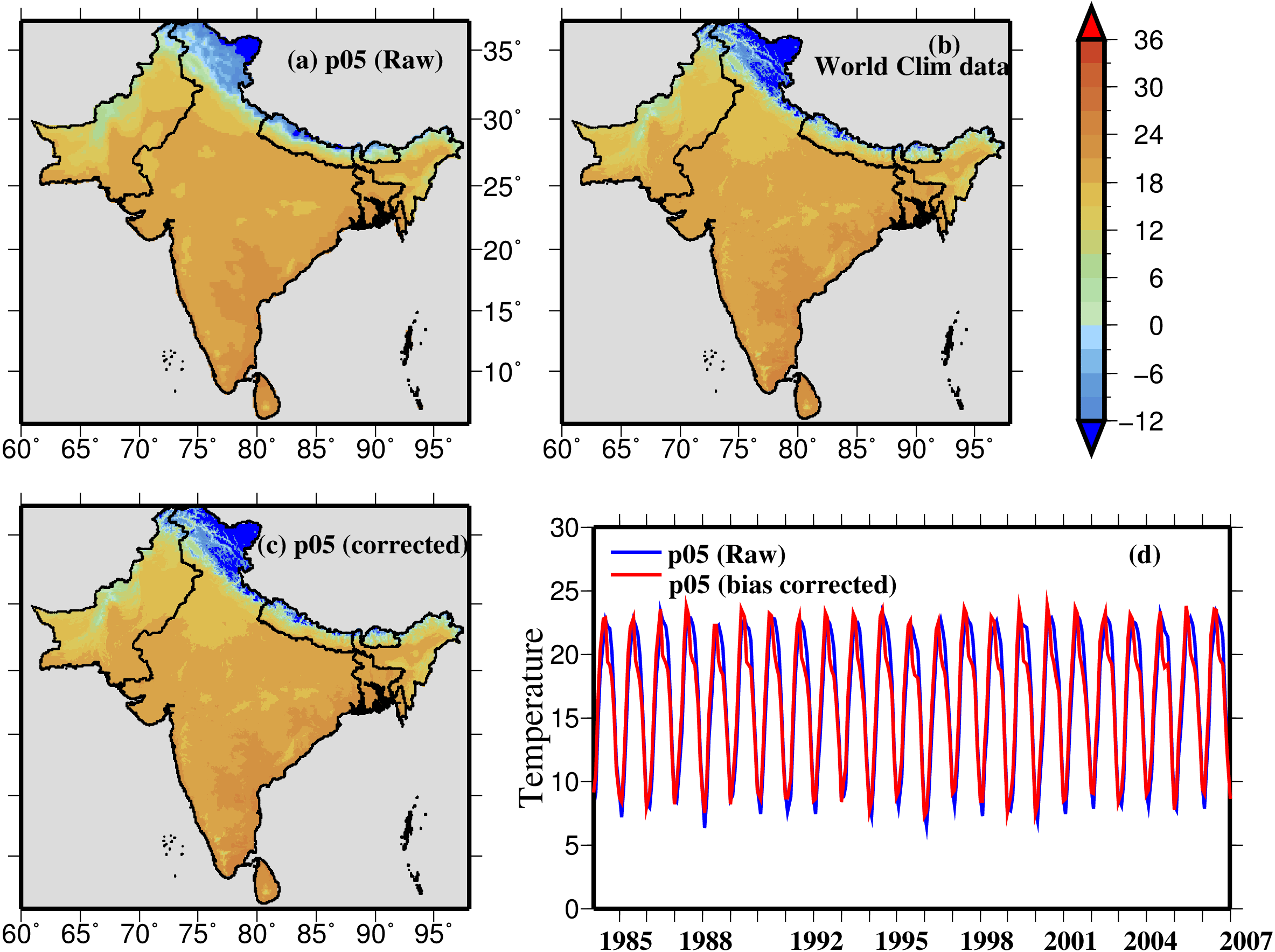


Figure S15 Bias-correction of minimum temperature from Global Ensemble Forecast System (GEFS). (a) Mean annual minimum temperature ( °C) at 0.05° spatial resolution (regridded from corrected GEFS 0.25°) for the period 1985-2007, (b) mean annual minimum temperature from the Worldclim data, (c) corrected mean annual minimum temperature at 0.05° spatial resolution against the Worldclim data for the period 1985-2007, and (d) mean monthly minimum temperature averaged over the South Asia from raw and corrected minimum temperature.


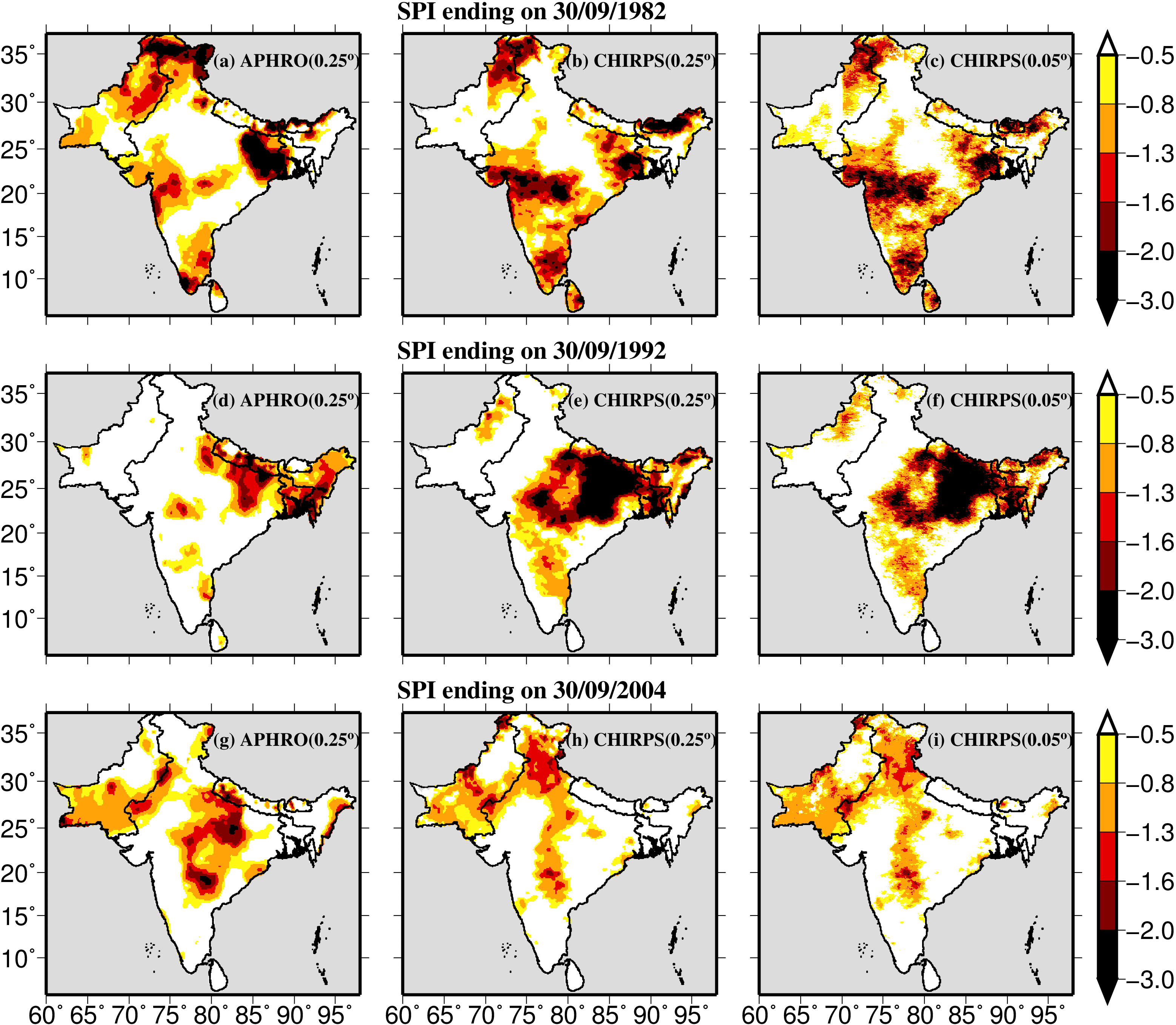


Figure S16. Meteorological drought using APHRODITE (0.25˚), bias-corrected CHIRPS (0.25˚), and bias-corrected CHIRPS (0.05˚) data. (a, b, c) 4-month SPI at the end of September for the year 1982; (d, e, f) 4-month SPI at the end of September for the year 1992; and (g, h, i) 4-month SPI at the end of September for the year 2004.


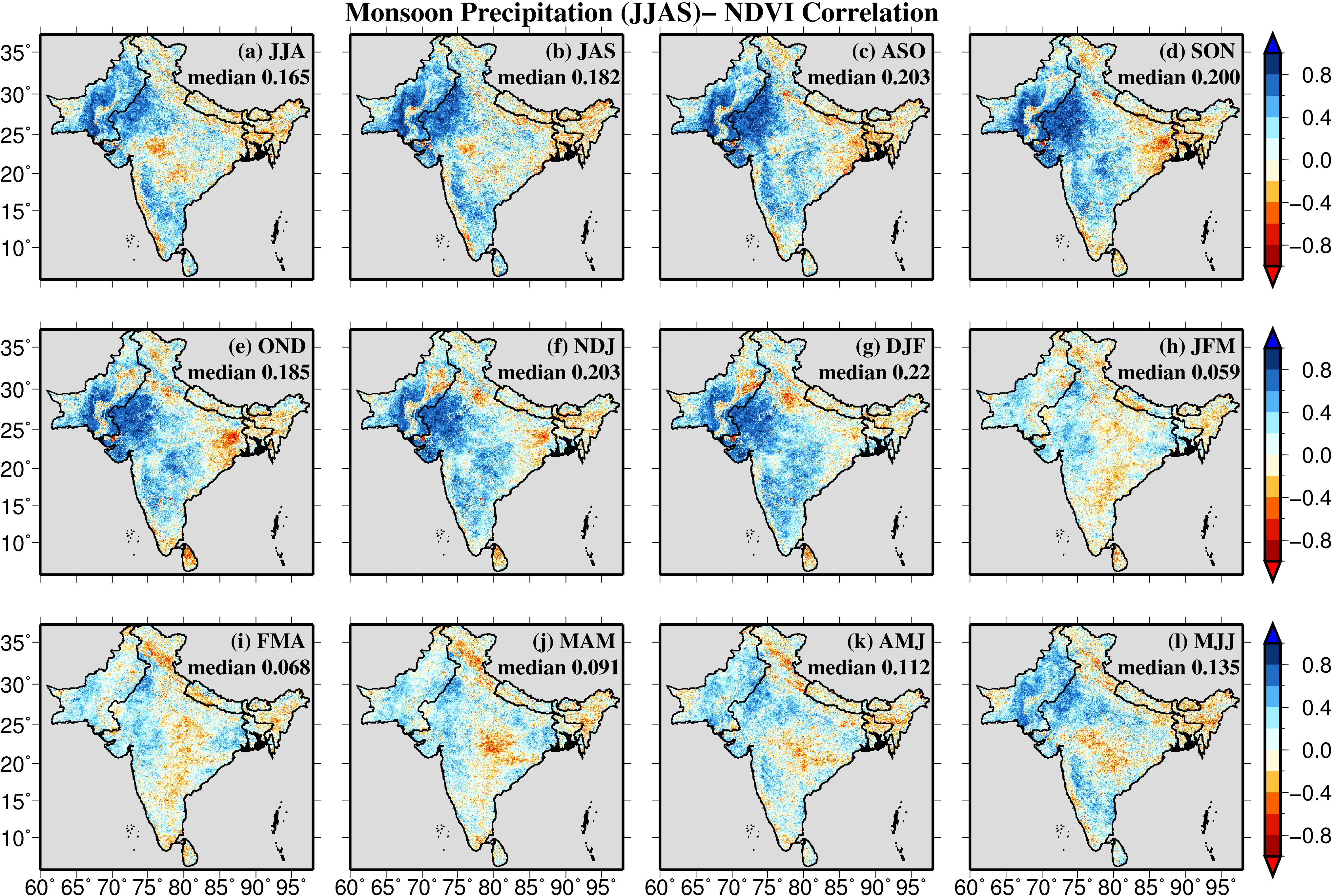


Figure S17. Correlation between 3-month averaged NDVI and the monsoon season (JJAS) rainfall for the period of 2000- 2015.


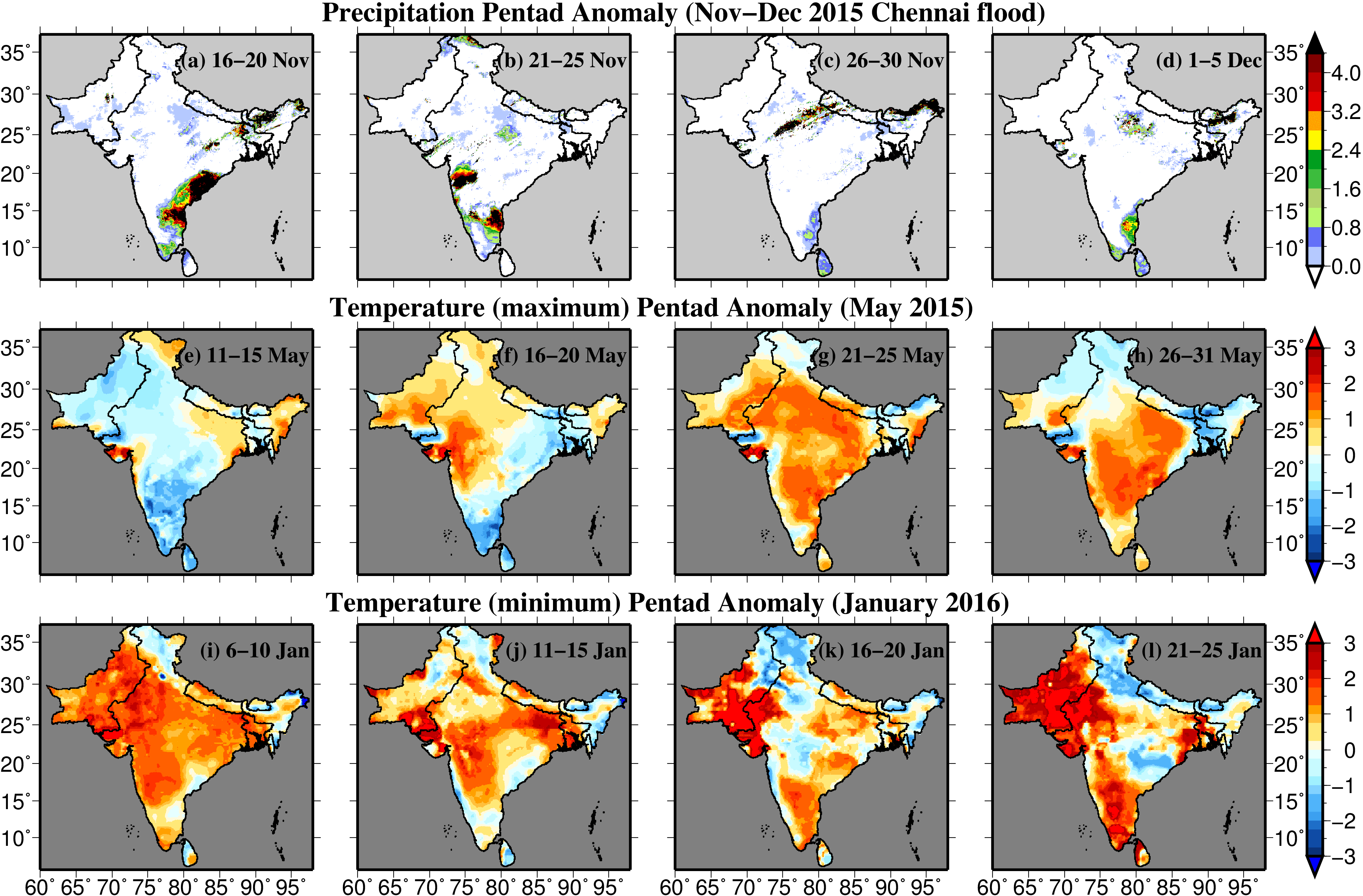


Figure S18 Application of high-resolution pentad data for climatic extremes. (a-d) extreme precipitation, (e-h) extreme heat event, and (i-l) extreme cold event.

**Table S1 Comparison of performance of the linear scaling and distribution mapping for bias correction in precipitation.**

| **Nash- Sutcliffe Efficiency** | | | |
| --- | --- | --- | --- |
|  |  |  |  |
| **Region** | **Before correction** | **After correction** | **After correction** |
|  |  | **(Linear Scaling method)** | **(Distribution Mapping method)** |
| 1 | 0.9142 | 0.9625 | 0.9681 |
| 2 | 0.9228 | 0.9533 | 0.9605 |
| 3 | 0.9430 | 0.9822 | 0.9849 |
| 4 | 0.8004 | 0.8512 | 0.8755 |
| 5 | 0.9425 | 0.9834 | 0.9835 |
| **Root Mean Square Error (RMSE)** | | | |
|  |  |  |  |
| **Region** | **Before correction** | **After correction** | **After correction** |
|  |  | **(Linear Scaling method)** | **(Distribution Mapping method)** |
| 1 | 20.7268 | 13.6988 | 12.6426 |
| 2 | 9.1083 | 7.0841 | 6.5190 |
| 3 | 26.0856 | 14.5739 | 13.4112 |
| 4 | 12.3498 | 10.6615 | 9.7532 |
| 5 | 22.2884 | 11.9747 | 11.9391 |
| **Correlation** | | | |
|  |  |  |  |
| **Region** | **Before correction** | **After correction** | **After correction** |
|  |  | **(Linear Scaling method)** | **(Distribution Mapping method)** |
| 1 | 0.9808 | 0.9852 | 0.9877 |
| 2 | 0.9968 | 0.9975 | 0.9975 |
| 3 | 0.9901 | 0.9971 | 0.9971 |
| 4 | 0.9936 | 0.9958 | 0.9962 |
| 5 | 0.9902 | 0.9945 | 0.9949 |

**Table S2 Same as Table S1 but for maximum temperature**

| **Nash- Sutcliffe Efficiency** | | | |
| --- | --- | --- | --- |
|  |  |  |  |
| **Region** | **Before correction** | **After correction** | **After correction** |
|  |  | **(Linear Scaling method)** | **(Distribution Mapping method)** |
| 1 | 0.1027 | 0.9512 | 0.9649 |
| 2 | 0.8519 | 0.9950 | 0.9945 |
| 3 | -0.9541 | 0.9714 | 0.9727 |
| 4 | -2.1349 | 0.9923 | 0.9917 |
| 5 | 0.7932 | 0.9860 | 0.9883 |
| **Root Mean Square Error (RMSE)** | | | |
|  |  |  |  |
| **Region** | **Before correction** | **After correction** | **After correction** |
|  |  | **(Linear Scaling method)** | **(Distribution Mapping method)** |
| 1 | 2.2644 | 0.5282 | 0.4475 |
| 2 | 2.0015 | 0.3693 | 0.3853 |
| 3 | 3.9880 | 0.4825 | 0.4714 |
| 4 | 10.8115 | 0.5353 | 0.5551 |
| 5 | 2.0452 | 0.5316 | 0.4865 |
| **Correlation** | | | |
|  |  |  |  |
| **Region** | **Before correction** | **After correction** | **After correction** |
|  |  | **(Linear Scaling method)** | **(Distribution Mapping method)** |
| 1 | 0.9826 | 0.9770 | 0.9839 |
| 2 | 0.9932 | 0.9975 | 0.9973 |
| 3 | 0.9675 | 0.9858 | 0.9878 |
| 4 | 0.9906 | 0.9962 | 0.9961 |
| 5 | 0.9847 | 0.9931 | 0.9943 |

**Table S3 Same as Table S1 but for minimum temperature**

| **Nash- Sutcliffe Efficiency** | | | |
| --- | --- | --- | --- |
|  |  |  |  |
| **Region** | **Before correction** | **After correction** | **After correction** |
|  |  | **(Linear Scaling method)** | **(Distribution Mapping method)** |
| 1 | -0.2568 | 0.9699 | 0.9728 |
| 2 | 0.9473 | 0.9950 | 0.9943 |
| 3 | 0.0739 | 0.9941 | 0.9930 |
| 4 | -1.7738 | 0.9915 | 0.9919 |
| 5 | 0.7485 | 0.9889 | 0.9890 |
| **Root Mean Square Error (RMSE)** | | | |
|  |  |  |  |
| **Region** | **Before correction** | **After correction** | **After correction** |
|  |  | **(Linear Scaling method)** | **(Distribution Mapping method)** |
| 1 | 2.7022 | 0.4182 | 0.3977 |
| 2 | 1.5239 | 0.4706 | 0.5007 |
| 3 | 4.6994 | 0.3741 | 0.4088 |
| 4 | 11.2391 | 0.6219 | 0.6065 |
| 5 | 2.8571 | 0.5993 | 0.5964 |
| **Correlation** | | | |
|  |  |  |  |
| **Region** | **Before correction** | **After correction** | **After correction** |
|  |  | **(Linear Scaling method)** | **(Distribution Mapping method)** |
| 1 | 0.9808 | 0.9852 | 0.9877 |
| 2 | 0.9968 | 0.9975 | 0.9975 |
| 3 | 0.9901 | 0.9971 | 0.9971 |
| 4 | 0.9936 | 0.9958 | 0.9962 |
| 5 | 0.9902 | 0.9945 | 0.9949 |
